# Two Novel Human Cytomegalovirus NK Cell Evasion Functions Target MICA for Lysosomal Degradation

**DOI:** 10.1371/journal.ppat.1004058

**Published:** 2014-05-01

**Authors:** Ceri A. Fielding, Rebecca Aicheler, Richard J. Stanton, Eddie C. Y. Wang, Song Han, Sepehr Seirafian, James Davies, Brian P. McSharry, Michael P. Weekes, P. Robin Antrobus, Virginie Prod'homme, Fabien P. Blanchet, Daniel Sugrue, Simone Cuff, Dawn Roberts, Andrew J. Davison, Paul J. Lehner, Gavin W. G. Wilkinson, Peter Tomasec

**Affiliations:** 1 Section of Medical Microbiology, Institute of Infection and Immunity, School of Medicine, Cardiff University, Cardiff, United Kingdom; 2 Cambridge Institute for Medical Research (CIMR), Wellcome Trust/MRC Building, Addenbrooke's Hospital, Cambridge, United Kingdom; 3 MRC-University of Glasgow Centre for Virus Research, Glasgow, United Kingdom; University of Alabama at Birmingham, United States of America

## Abstract

NKG2D plays a major role in controlling immune responses through the regulation of natural killer (NK) cells, αβ and γδ T-cell function. This activating receptor recognizes eight distinct ligands (the MHC Class I polypeptide-related sequences (MIC) A andB, and UL16-binding proteins (ULBP)1–6) induced by cellular stress to promote recognition cells perturbed by malignant transformation or microbial infection. Studies into human cytomegalovirus (HCMV) have aided both the identification and characterization of NKG2D ligands (NKG2DLs). HCMV immediate early (IE) gene up regulates NKGDLs, and we now describe the differential activation of ULBP2 and MICA/B by IE1 and IE2 respectively. Despite activation by IE functions, HCMV effectively suppressed cell surface expression of NKGDLs through both the early and late phases of infection. The immune evasion functions UL16, UL142, and microRNA(miR)-UL112 are known to target NKG2DLs. While infection with a UL16 deletion mutant caused the expected increase in MICB and ULBP2 cell surface expression, deletion of UL142 did not have a similar impact on its target, MICA. We therefore performed a systematic screen of the viral genome to search of addition functions that targeted MICA. US18 and US20 were identified as novel NK cell evasion functions capable of acting independently to promote MICA degradation by lysosomal degradation. The most dramatic effect on MICA expression was achieved when US18 and US20 acted in concert. US18 and US20 are the first members of the US12 gene family to have been assigned a function. The US12 family has 10 members encoded sequentially through US12–US21; a genetic arrangement, which is suggestive of an ‘accordion’ expansion of an ancestral gene in response to a selective pressure. This expansion must have be an ancient event as the whole family is conserved across simian cytomegaloviruses from old world monkeys. The evolutionary benefit bestowed by the combinatorial effect of US18 and US20 on MICA may have contributed to sustaining the US12 gene family.

## Author Summary

Human cytomegalovirus (HCMV) is a herpesvirus that infects most people in the world, usually without producing symptoms. However, infection is life-long and must be kept in check by the immune system. When the immune system is weakened, the outcome of HCMV infection can be very serious. Thus, HCMV is the major cause of birth defects resulting from infection of the fetus during pregnancy, and it can cause severe disease in people with a weakened immune system, especially transplant recipients and HIV/AIDS patients. One type of immune cell, the natural killer (NK) cell, is crucial in controlling cells in the body that are abnormal. They do this by recognizing cells, which have special stress proteins on their surface, and killing them. When cells are infected with HCMV, they start to make these stress proteins. However, the virus has evolved ways to stop NK cells from killing infected cells by quickly stopping the stress proteins from reaching the surface. We have now identified two HCMV genes that target a major stress protein (called MICA) and cause its rapid destruction. Removing these two genes from HCMV renders infected cells very susceptible to killing by NK cells. This discovery might help the development of new ways to fight HCMV.

## Introduction

Human cytomegalovirus (HCMV) is a clinically important pathogen, which is particularly associated with high levels of morbidity and mortality in immuno-compromised individuals. Systemic HCMV infection results in a higher incidence of graft rejection in transplant recipients and a wide range of end-organ disease including pneumonia, enteritis, hepatitis and retinitis (specifically in HIV-AIDS). The virus is the major cause of congenital birth defects, with long-term sequelae including mental retardation and sensorineural hearing loss [1]. A correlation has been established between infections and two common and aggressive brain tumors (medulloblastoma and glioblastoma multiforme) [2], [3], which remains controversial [4], while HCMV has also been implicated in cardiovascular disease, arthritis and in imprinting characteristic changes on the immune repertoire [2], [5], [6]. Nevertheless, the vast majority of HCMV primary infections are subclinical and are followed by life-long asymptomatic persistence. Thus, while a competent immune response is unable to eliminate this herpesvirus, in most individuals it is effective at limiting virus replication and preventing disease.

HCMV has the largest genome (∼236 kbp) of any characterized human virus and is a paradigm of viral immune evasion. A substantial proportion of its coding capacity is dedicated to evading or modulating immune defenses, and includes genes that target the antigen presentation and processing pathway (US2, US3, US6, US11, and miR US4-1) [7], [8], [9], [10], [11], [12], [13], human leukocyte antigen (HLA)-G (US10) [14], T-cell receptor signaling (UL11) [15], TNF-related apoptosis-inducing ligand (TRAIL) death receptor signaling (UL141) [16], interferon signaling or its downstream effects (UL83) [17], [18], [19] and dendritic cell cytokine secretion (UL7) [20]. The virus also encodes interleukin-10 (UL111A) [21] and interleukin-8 (UL146) homologs [22]. Natural killer (NK) cells play a critical role in the control of HCMV infections; individuals with genetic defects in their NK cell response exhibit extreme susceptibility to the virus [23], [24], [25], [26]. The prognosis of bone marrow and renal transplant patients infected with HCMV infection has recently been demonstrated to correlate with the genotype of specific NK cell receptors (KIR) [27]. The significance of the NK cell response is also reflected in the great lengths to which HCMV goes to evade it. To date, UL16, UL18, UL40, UL83, UL141, UL142, and miR-UL112 have all been identified as NK cell evasion functions [28], [29], [30], [31], [32], [33], [34], [35], [36], [37], [38], [39], [40].

NK cells constitute a heterogeneous population that express a mosaic of inhibitory and activating receptors, each capable of recognizing and responding to specific ligands presented by potential target cells [41]. The activating receptor NKG2D is remarkable in being expressed ubiquitously on all NK cells and capable of recognizing at least 8 distinct ligands: the major histocompatibility complex class I (MHC-I) chain-related molecules (MICA and MICB) and the UL16-binding proteins 1–6 (ULBP1-ULBP6) [41], [42], [43]. NKG2D ligands (NKG2DL) can be induced on the cell surface during times of cellular stress, including genotoxic damage, growth stimulation or viral infection. Within 24 h post infection (p.i.), HCMV activates all NKG2DL with the exception of ULBP4 [44]. More specifically, the HCMV major immediate early (IE) proteins IE1 and IE2 (encoded by genes UL123 and UL122, respectively) have been implicated in activating transcription of the MICA/B promoters [45]. HCMV counters this up regulation through (i) the sequestration of MICB, ULBP1, ULBP2, and ULBP6 in the ER by the UL16 protein [33], [34], [35], [36], [43], [46], (ii) the retention of MICA and ULBP3 by the UL142 protein within the *cis*-Golgi [32], [47], [48], and (iii) the microRNA miRUL112 targeting the MICB transcript [28]. The high degree of sequence polymorphism exhibited by both MICA (80 alleles) and MICB (33 alleles) has the potential to provide an added challenge to the virus. Indeed, it has been proposed that this degree of MICA/B diversity may have been selected as a mechanism by which cells evade HCMV infection [48].

We sought initially to explore whether the rapid kinetics of NKG2DL expression during HCMV infection provides a window of opportunity for NK cell recognition prior to the expression of virus-encoded immune evasion proteins. In characterising the up regulation of NKG2DL, we identified a differential effect on MICA and MICB by the HCMV IE gene products, and yet HCMV immune evasion functions were effective in preventing surface expression of these NKG2DL through the early phase of lytic infection. However, the full complement of HCMV immune evasion functions targeting the NKG2DL had not been defined. We now describe the identification and characterization of two novel NK cell evasion genes, HCMV US18 and US20 act individually and in concert to suppress expression cell surface MICA. The US12 gene family comprise some 10 genes arranged sequentially (US12-US21) and uninterrupted through a 9 kb stretch if the U_S_ genomic region [49]. US18 and US20 are the first members of this large gene family to be assigned a function.

## Results

### Differential regulation of NKG2DL by the HCMV major IE genes

**Figure 1:**
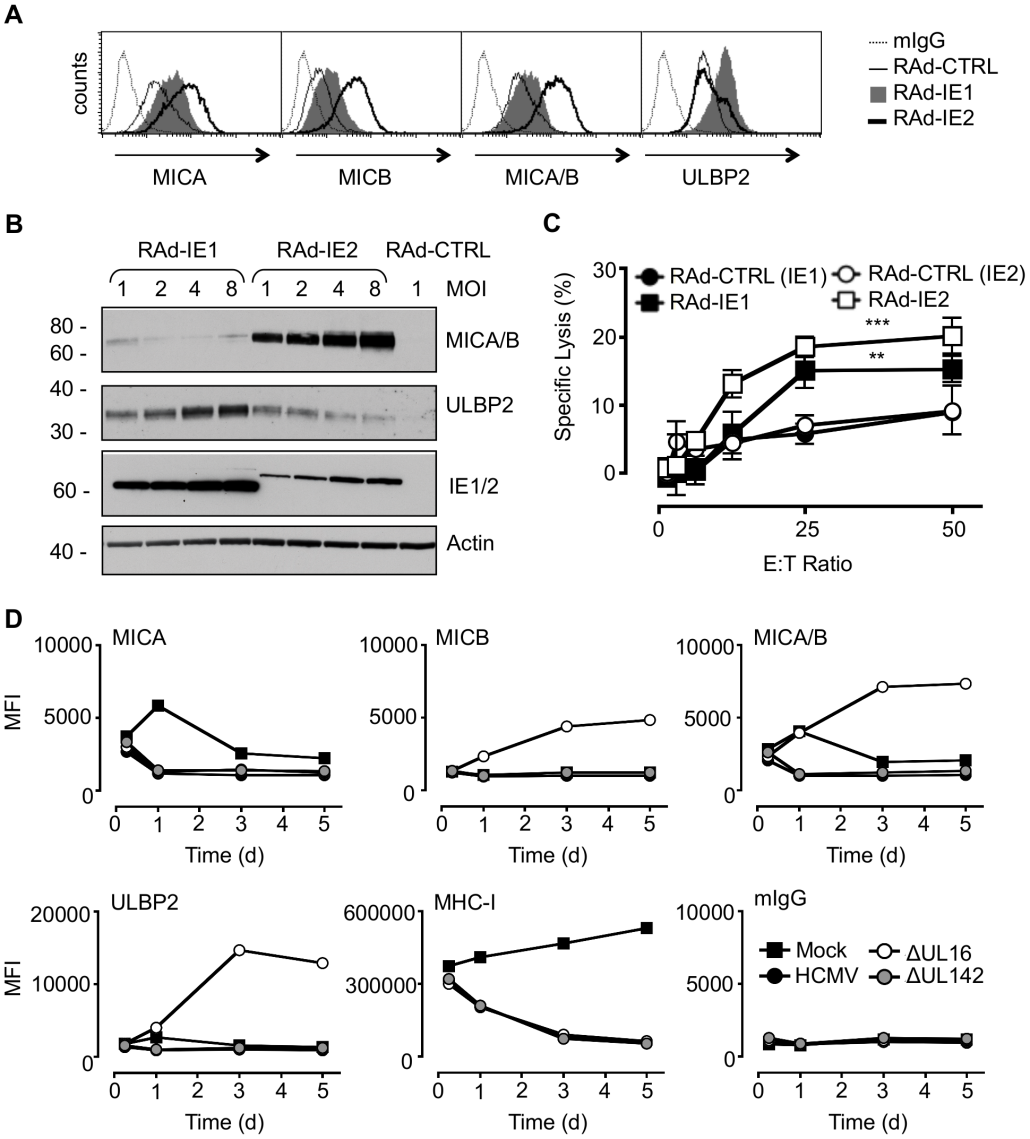
Regulation of NKG2DL during HCMV infection. A. Fibroblasts (HF-CARs) were infected with RAds expressing IE1 or IE2 at an m.o.i. of 1. Cell surface expression of MICA, MICB, MICA/B, and ULBP2 were analyzed by flow cytometry at 72 h p.i. The results shown are representative of 2 independent experiments. B. Fibroblasts were infected with RAds expressing IE1 or IE2 at an m.o.i. of 1–8 or RAd control (CTRL) moi 1. Expression of MICA, MICB, MICA/B, and ULBP2 was analyzed by immunoblotting at 72 h p.i. The results shown are representative of 2 independent experiments. C. Fibroblasts were infected with RAds expressing IE1 or IE2 or RAds lacking an insert (RAd-CTRL). Infected cells were used as targets for IFN-α treated NK cells in a ^51^Cr release assay at an E∶T ratio of 1∶50. The results shown are representative of 2 independent experiments and were analyzed by 2-way ANOVA (** *p*<0.01, *** *p* = 0.001). D. Fibroblasts (HF-TERTs) were mock-infected or infected with HCMV strain Merlin or deletion mutants of this strain lacking UL16 (ΔUL16) or UL142 (ΔUL142). Cell surface expression of MICA, MICB, MICA/B, ULBP2, MHC-I, or murine immunoglobulin (mIgG) was analyzed between 6 and 120 h p.i. by flow cytometry. The results are shown as median fluorescence intensity (MFI) and are representative of 2 independent experiments.

The regulation and function of NKG2DL exhibits the properties of an intrinsic host immune defense system designed to sense cellular changes associated with danger or pathogen-associated molecular patterns. The existence of eight dedicated ligands provides great scope for the NKG2D receptor to sense and react to a broad range of stimuli. HCMV IE genes have previously been implicated in the transcriptional activation of NKG2Ls [45], [50]. Therefore, we first examined the capacity of IE1 and IE2 to regulate the various NKG2DL. When expressed using adenovirus (Ad) vectors, IE1 induced relatively modest increases in MICA and MICB, but provided for a major up regulation in ULBP2 both at the level of total protein expression and specifically on the cell surface (Fig. 1A and B). In contrast, IE2 induced strong activation of MICA and MICB, yet only a small increase ULBP2 levels. IE1 and IE2 were thus found to differentially activate individual ligands recognized by the NKG2D activating receptor. Consistent with their capacity to up regulate NKG2DL, IE1 and IE2 were each able individually to sensitize cells to NK cell-mediated cytotoxicity (Fig. 1C). We concluded that the infected cell responds to differential sensing of the expression of IE1 and IE2 by promoting the up regulation of ULBP2 and MICA/B, respectively.

### Additional HCMV functions implicated in regulating MICA

We hypothesized that HCMV-infected cells may be vulnerable to NK cell surveillance during the early phase if there were a temporal window between the activation of NKG2Ls by IE1 and IE2 and the expression of effective HCMV counter measure. To investigate this possibility, the expression of MICA, MICB, ULBP1, ULBP2 and ULBP3 was monitored throughout the course of productive HCMV infection (Fig. 1D, Fig. S1, Fig. S2). MHC Class-I (MHC-I) expression was included as an infection control. At 6 h p.i., there was no difference between mock- and virus-infected cells in MHC-I or NKG2DL expression. However, cell surface down-regulation of MHC-I expression was clearly observed through the early and late phases of a productive replication cycle (24 to 120 h p.i). At no stage during the replication cycle did HCMV infection induce an up regulation MICA, MICB, or ULBP2 expression at the cell surface. Thus, we were unable to detect the hypothesized temporal window during which the infected cell may be vulnerable to NK cytotoxicity through activation of NKG2D.

As the NKG2DLs must first respond to IE gene expression, they should be activated with similar kinetics to those of a standard HCMV-encoded early gene. To be effective, the corresponding HCMV counter measure must therefore also be expressed with early phase kinetics. UL16 is clearly essential during this process, as its deletion (strain Merlin ΔUL16) increased cell surface MICB, ULBP1 and ULBP2 levels as early as 24 h p.i. (Fig. 1D, Fig. S1B, Fig. S2). In contrast, deletion of UL142 (ΔUL142) had no overt effect on the expression of any NKG2DL tested, including MICA and ULBP3 (Fig. 1D, Fig. S1B, Fig. S2). Moreover, fibroblasts infected with the high-passage HCMV laboratory strain AD169, which lacks UL142 and several other genes, showed a level of control of cell surface MICA (Fig. S3) comparable to that induced by strain Merlin (Fig. 1D, Fig. S1B). The efficient control of MICA expression in cells infected with strain AD169 and strain Merlin ΔUL142 implied the existence of additional HCMV functions capable of targeting MICA. We concluded that, although HCMV activates MICA cell surface expression, this response is counteracted in the context of productive HCMV infection by the action of a previously uncharacterized function.

### US18–US22 is a major HCMV locus controlling cell surface expression of MICA

**Figure 2:**
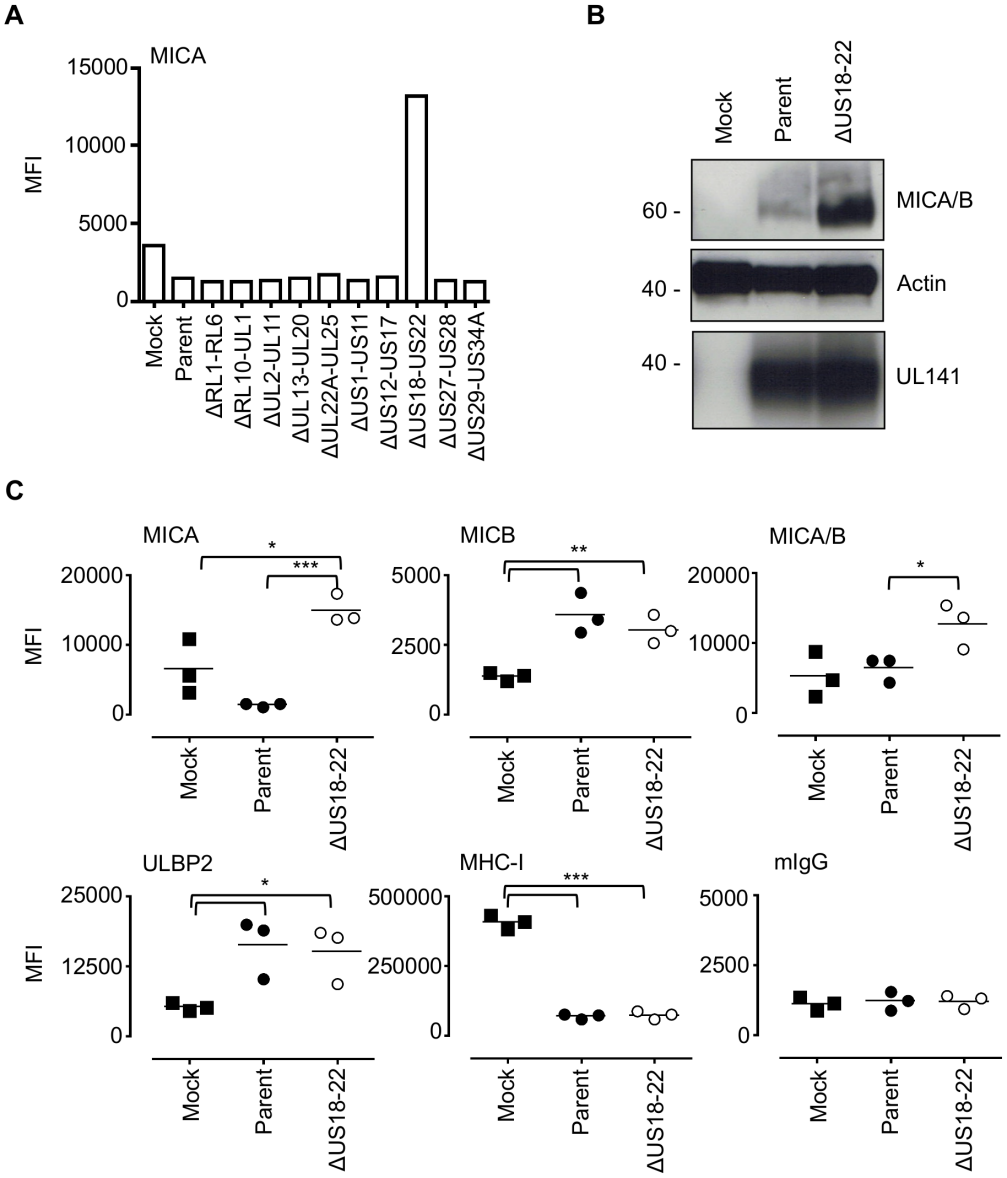
The HCMV gene function targeting MICA maps to the US18–22 gene locus. A. Fibroblasts (HF-TERTs) were mock infected or infected with HCMV parent virus or a series of ‘block’ deletion mutants for 72 h. Cell surface expression of MICA was analyzed by immunostaining and flow cytometry. Results shown are the median fluorescence intensity (MFI) and are representative of two independent experiments. B. Fibroblasts (HF-TERTs) were mock infected or infected with HCMV parent virus or a deletion mutant lacking US18–22 (ΔUS18–22) for 72 h. Hydrophobic proteins were extracted using Triton X-114 and protein expression analyzed by immunoblotting. Data shown is representative of three independent experiments. C. Fibroblasts (HF-TERTs) were mock infected or infected with HCMV or a deletion mutant lacking US18–22 (ΔUS18–22) for 72 h. Cell surface expression was analyzed by immunostaining and flow cytometry. Results shown are the median fluorescent intensity (MFI) and from three independent experiments. The mean of the results was analysed by Student's t-test (*P<0.05, **P<0.01, ***P<0.001).

HCMV contains 170 canonical protein-coding genes, of which only 45 are essential for replication in fibroblast cells *in vitro* [51], [52], [53]. In order to map HCMV functions to particular parts of the genome, we generated 10 mutants in which blocks of non-essential genes totaling 56 genes were deleted (Fig. S4A). To facilitate detection of novel NK evasion functions, these mutants were generated on a strain Merlin background that already lacked UL16 and UL18 (ΔUL16ΔUL18) and contained a UL32-GFP fusion reporter. Screening cells infected with these block deletion mutants identified one mutant lacking US18–22 (ΔUS18–22), which led to a marked increase in cell surface MICA detected by flow cytometry (Fig. 2A). This mutant was also found to replicate less efficiently than the parent virus (data not shown). Infection with this mutant also resulted in a very marked increase in the total cellular abundance of MICA/B (Fig. 2B). Specific antibodies to other NKG2DLs were then used to see if this effect was specific by flow cytometry. As the block deletion mutants were made on a ΔUL16ΔUL18 background, the parental HCMV control up regulated MICB and ULBP2 relative to mock-infected cells (Fig. 2C, Fig. S4B), whereas the ΔUS18–22 mutant up regulated cell surface levels of MICA (Fig. 2C, Fig. S4B) relative to both mock- and parental HCMV-infections. These results suggest the potential for an HCMV NK cell evasion function that acts by promoting degradation of an NKG2DL.

### US18 and US20 down-regulate MICA

**Figure 3:**
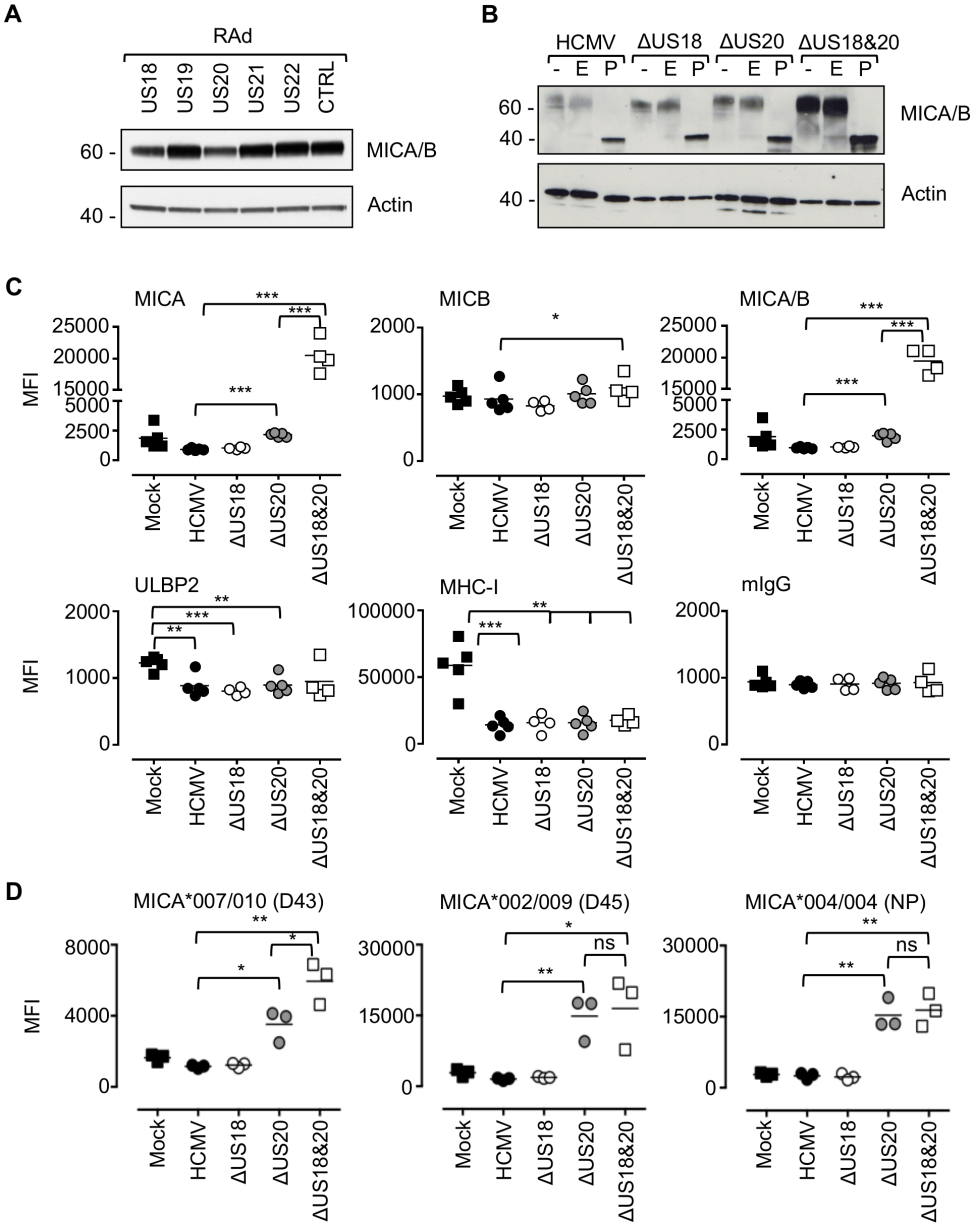
US18 and US20 down-regulate MICA levels. A. Fibroblasts (HF-CARs) were infected with recombinant adenovirus expressing the individual US18–US22 genes for 72 h. Hydrophobic proteins were extracted using Triton X-114 and protein expression analyzed by immunoblotting. Data shown is representative of four similar independent experiments. B. Fibroblasts (HF-TERTs) were mock infected or infected with HCMV, ΔUS18, ΔUS20, ΔUS18&20 for 72 h. Hydrophobic proteins were extracted using Triton X-114, mock-treated (-) or treated with either Endoglycosidase H (E) or PNGase F (P) and protein expression analyzed by immunoblotting. Results are representative of two independent experiments. C. Fibroblasts (HF-TERTs) were mock infected or infected with HCMV, ΔUS18 or ΔUS18, ΔUS20 for 72 h. Cell surface expression was analyzed by immunostaining and flow cytometry. Results shown are the median fluorescence intensity (MFI) and are from four independent experiments. The mean of the results was analysed by Student's t-test (*P<0.05, **P<0.01, ***P<0.001). *D.* Dermal fibroblasts expressing different MICA alleles were mock infected or infected with HCMV, ΔUS18 or ΔUS18, ΔUS20 for 72 h. Cell surface expression was analyzed by immunostaining and flow cytometry. Results shown are the median fluorescence intensity (MFI) and are from three independent experiments. The mean of the results was analysed by Student's t-test (*P<0.05, **P<0.01, ns = not significant).

To map the MICA-suppressing function more precisely, the 5 genes in the US18–US22 region were expressed individually in fibroblasts using an Ad vector, and expression monitored by detection of the V5 tag by intracellular flow cytometry (Fig. S5). Expression of US18 or US20 led to reductions in MICA/B levels by immunoblotting (Fig. 3A). Interestingly, expression of the US18–US22 genes individually was each associated with a marginal reduction in MICA cell surface expression; thus genes adjacent to US18 and US20 could also be contributing to the suppression of MICA (Fig. S6). In the context of an HCMV infection, deletion of US18 and US20 led to an increase MICA/B levels as monitored by immunoblotting (Fig. 3B). Deletion of both US18 and US20 led to a further increase in MICA/B levels (Fig. 3B). No differences in MICA/B glycosylation were observed in cells infected with the different HCMV US18 and US20 deletion mutants when samples were treated with Endoglycosidase H/EndoH (E) or Peptide N-Glycosidase F/PNGase F (P) (Fig. 3B). Almost all of the MICA/B was EndoH-resistant and therefore consistent with its localization in a post-ER compartment (e.g. cis-Golgi, lysosomes, or cell surface expression).

By flow cytometry, deletion of US18 alone had no discernible effect on cell surface expression of MICA, MICB, ULBP2 or MHC-I (Fig. 3C, Fig. S7). In contrast, deletion of US20 induced a modest but significant increase in surface levels of MICA compared to parental virus, but MICB, ULBP2 and MHC-I expression were unchanged. However, deletion of both US18 and US20 caused a much more dramatic increase in MICA levels, similar to that observed for US18–22 block deletion mutant (Fig. 2C and 3C). These mutants had no appreciable difference in replication efficiency (data not shown). Thus, absence of either US18 or US20 was partially compensated by the other gene, whereas absence of both genes had a more than additive effect on MICA expression.

The HF cell lines (HF-TERT and HF-CAR) were immortalized lines derived from the same donor HFs, which have a MICA genotype, MICA*016/027 [54]. The same pattern of MICA cell surface expression was obtaining using the primary parent HF cell line infected with the US18 and US20 deletion mutants (data not shown). To determine whether US18 and US20 could target a range of MICA alleles, we performed a similar analysis in a number of donor dermal fibroblast cell lines with different MICA genotypes. These experiments revealed a greater dependence on US20 for regulating MICA, as there was little (D43, MICA*007/010) or no difference (D45, MICA*002/009 and NP, MICA*004/004) between the single US20 deletion mutant and US18 and US20 double deletion mutant (Fig. 3D, Fig. S8).

### Analysis of US18 and US20 function in the context of HCMV

**Figure 4:**
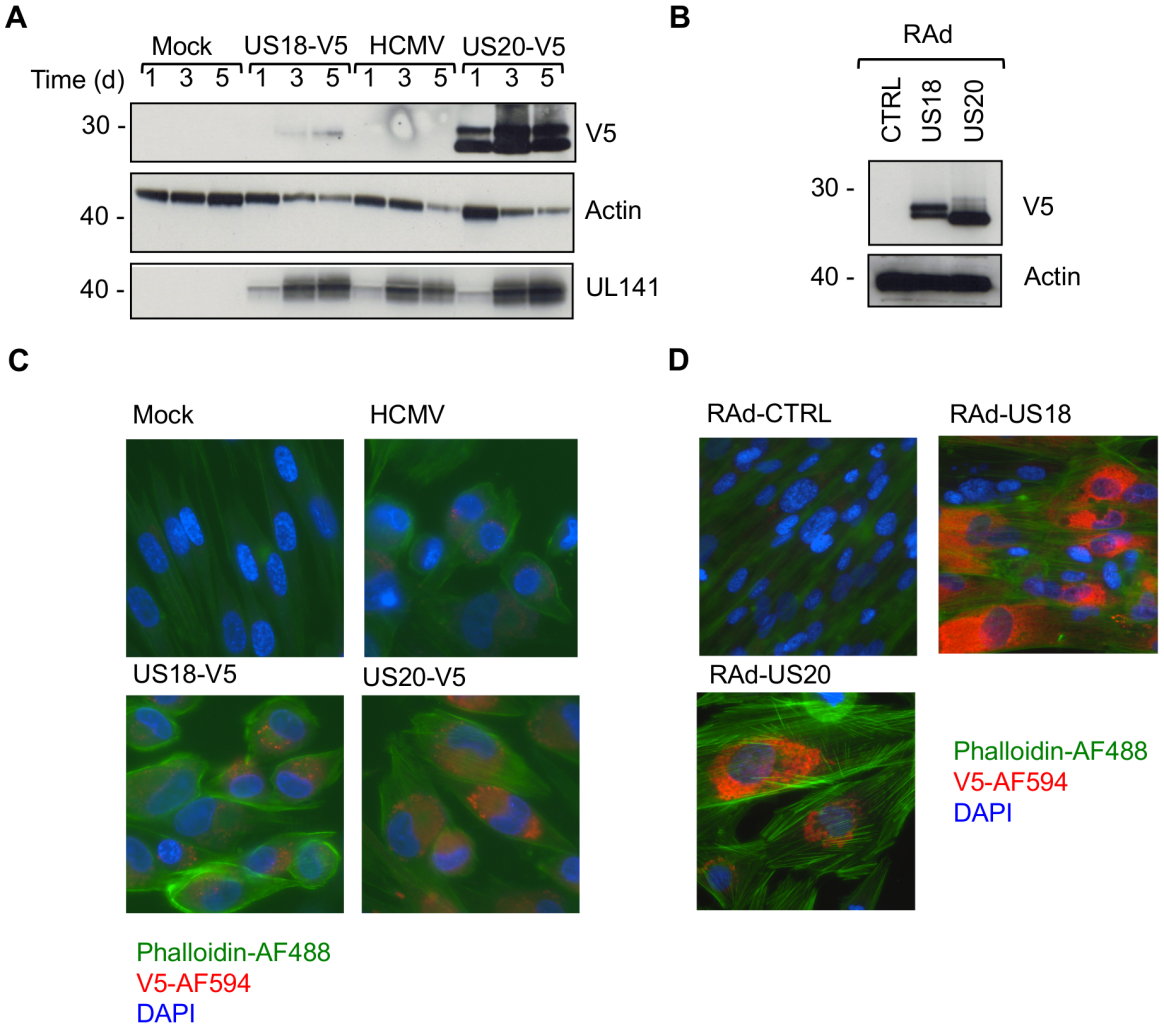
Comparison of US18 and US20 expressed during HCMV infection and by adenovirus expression vectors. A. HF-TERTs were either mock-infected or infected with HCMV wt or HCMV with V5-tagged US18 and US20 genes for 1, 3 or 5 days at an m.o.i. of 10. Cells were harvested and hydrophobic proteins extracted with Triton-X114 before immunoblotting with an anti-V5, actin or UL141 antibodies. Results are representative of two independent experiments. B. HF-CARs were infected with adenovirus control or expressing US18 or US20. Cells were harvested and hydrophobic proteins extracted with Triton-X114 before immunoblotting with an anti-V5 and actin antibodies. Results are representative of five independent experiments. C. HF-TERTs were either mock-infected or infected with HCMV wt or HCMV with V5-tagged US18 and US20 genes for 3 days at an m.o.i. of 10. Cells were fixed, permeabilized and immunostained with anti-V5 antibodies and anti-mouse Alexa Fluor-594 secondary antibody, then counterstained with the nuclear stain DAPI and actin-binding phalloidin-Alexa Fluor 488. Results are representative of three independent experiments. D. HF-CARs were infected with adenovirus control or expressing US18 or US20. Cells were fixed, permeabilized and immunostained with anti-V5 antibodies and anti-mouse Alexa Fluor-594 secondary antibody, then counterstained with the nuclear stain DAPI and actin-binding phalloidin-Alexa Fluor 488. Results are representative of six independent experiments.

To complement findings obtained with exogenous US18 and US20 gene expression, the equivalent C-terminal V5 antigenic tag was added to the genes in the context of the HCMV genome. In contrast to transcriptomic studies [52], pUS18-V5 expression was expressed at lower levels than pUS20-V5 at all stages of the HCMV replication cycle, as gauged by the intensity of staining from the V5 epitope tag (Fig. 4A). Therefore, functional differences between the US18 and US20 single HCMV deletion mutants (Fig. 3C) may reflect this difference in detectable expression levels, or perhaps pUS18 is subject to a more rapid cellular turnover than pUS20. However, comparable expression between pUS18 and pUS20 was observed when using Ad vectors (Fig. 4B). Analysis of pUS18 and pUS20 expression by immunofluorescence staining in the context of HCMV revealed that both proteins were located in subcellular punctate structures (Fig. 4C). This pattern of expression was similar to that observed using the US20 RAd (Fig. 4D), but contrasted with the ER-like expression pattern detected for the US18 RAd (Fig. 4D). We concluded that, whether expressed from Ads or in the context of HCMV, both proteins reside within an intracellular compartment from which they are able to regulate MICA expression.

### HCMV targets MICA via US18 and US20 for lysosomal degradation

**Figure 5:**
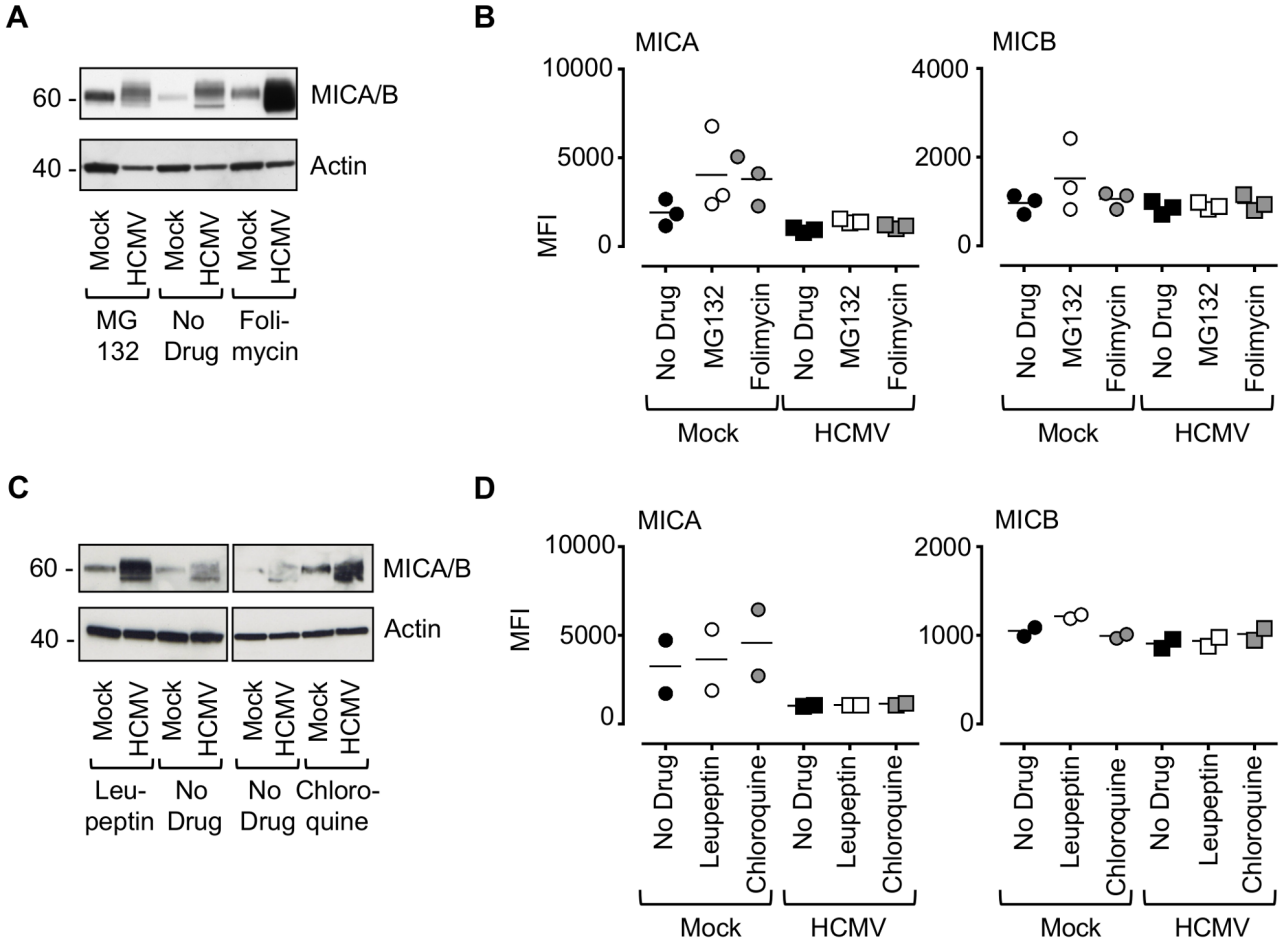
Effect of proteasomal and lysosomal inhibitors on MICA/B degradation by HCMV. A. Fibroblasts (HF-TERTs) were mock-infected or infected with HCMV for 72 h. A proteasomal inhibitor (MG132) or lysososomal inhibitor (folimycin) was added 12 h prior to harvesting. Hydrophobic proteins were extracted using Triton X-114, and protein expression was analyzed by immunoblotting. The results are representative of 3 independent experiments. B. Fibroblasts (HF-TERTs) were either mock infected or infected with HCMV for 72 h. A proteasomal inhibitor (MG132) or lysososomal inhibitor (folimycin) was added 12 h prior to harvesting. Cell surface expression was analyzed by immunostaining and flow cytometry. The results shown are the median fluorescent intensity (MFI) and are representative of 3 independent experiments. C. Fibroblasts (HF-TERTs) were mock-infected or infected with HCMV for 72 h. The lysosomal inhibitors leupeptin or chloroquine were added 12 h prior to harvesting. Hydrophobic proteins were extracted using Triton X-114, and protein expression was analyzed by immunoblotting. The results are representative of 2 independent experiments. D. Fibroblasts (HF-TERTs) were either mock infected or infected with HCMV for 72 h. The lysosomal inhibitors leupeptin or chloroquine were added 12 h prior to harvesting. Cell surface expression was analyzed by immunostaining and flow cytometry. The results shown are the median fluorescent intensity (MFI) and are representative of 2 independent experiments.

Next, we sought to determine the fate of MICA in HCMV-infected cells. Cells were infected with HCMV strain Merlin in combination with chemical inhibitors of the major protein degradation pathways that act via the proteasome (MG132) or lysosome (folimycin) (Fig. 5A and B, Fig. S9A). Inhibition of lysosomal, but not proteasomal, degradation greatly increased the cellular levels of MICA/B as assessed by immunoblotting (Fig. 5A). Addition of either MG132 or folimycin had no effect on cell surface MICA or MICB expression in either mock or HCMV-infected cells (Fig. 5B, Fig. S9A). Treatment with a further two lysosomal inhibitors, leupeptin (a protease inhibitor) and chloroquine (an inhibitor of lysosomal acidification) led to an increase in MICA/B levels by immunoblotting (Fig. 5C), but no effect on cell surface MICA or MICB expression by flow cytometry (Fig. 5D, Fig. S9B). Folimycin and chloroquine affect the acidification of the lysosomes and the Golgi apparatus, while leupeptin is a protease inhibitor targeting lysosomal proteases. Therefore, these inhibitors will prevent degradation of proteins within the lysosome by preventing the action of proteolytic enzymes requiring an acidic pH, but will not redirect them from this compartment to the surface. Therefore HCMV appears to target MICA for lysosomal degradation, and this protein is retained within the cell following lysosomal inhibition.

**Figure 6:**
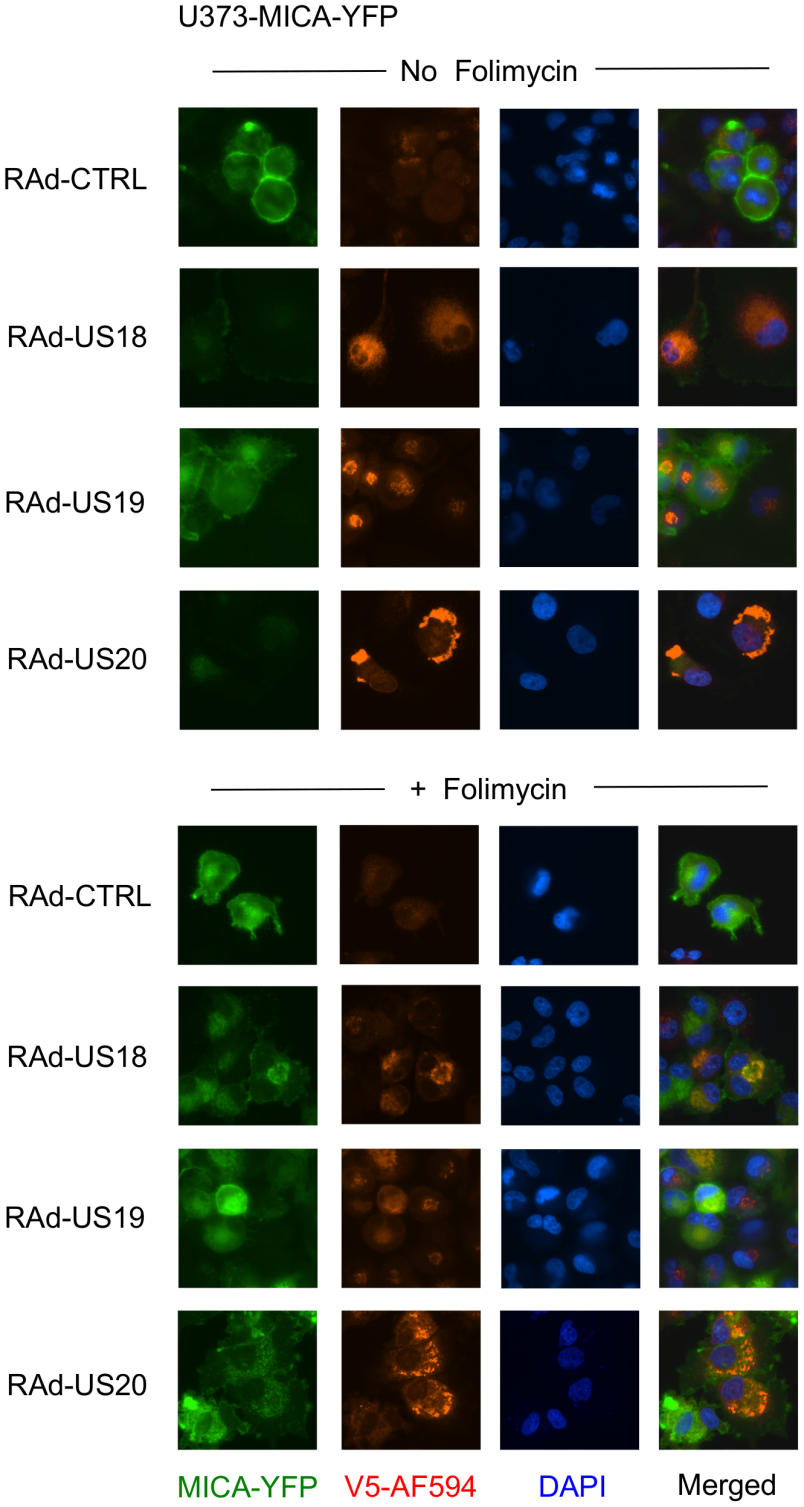
Degradation of MICA-YFP by US18 and US20 in U373 cells. U373 cells stably transfected with MICA-YFP were infected with control or US18-V5, US19-V5 or US20-V5 expressing adenovirus for 72 h in duplicate. Folimycin was added to one duplicate well 12 h prior to analysis. Cells were fixed and permeabilized and immunostained with a mouse antibody for the V5 epitope tag plus anti-mouse IgG-Alexa Fluor 594 secondary antibody and counterstained with the nuclear dye DAPI. Results are representative of three similar independent experiments.

**Figure 7:**
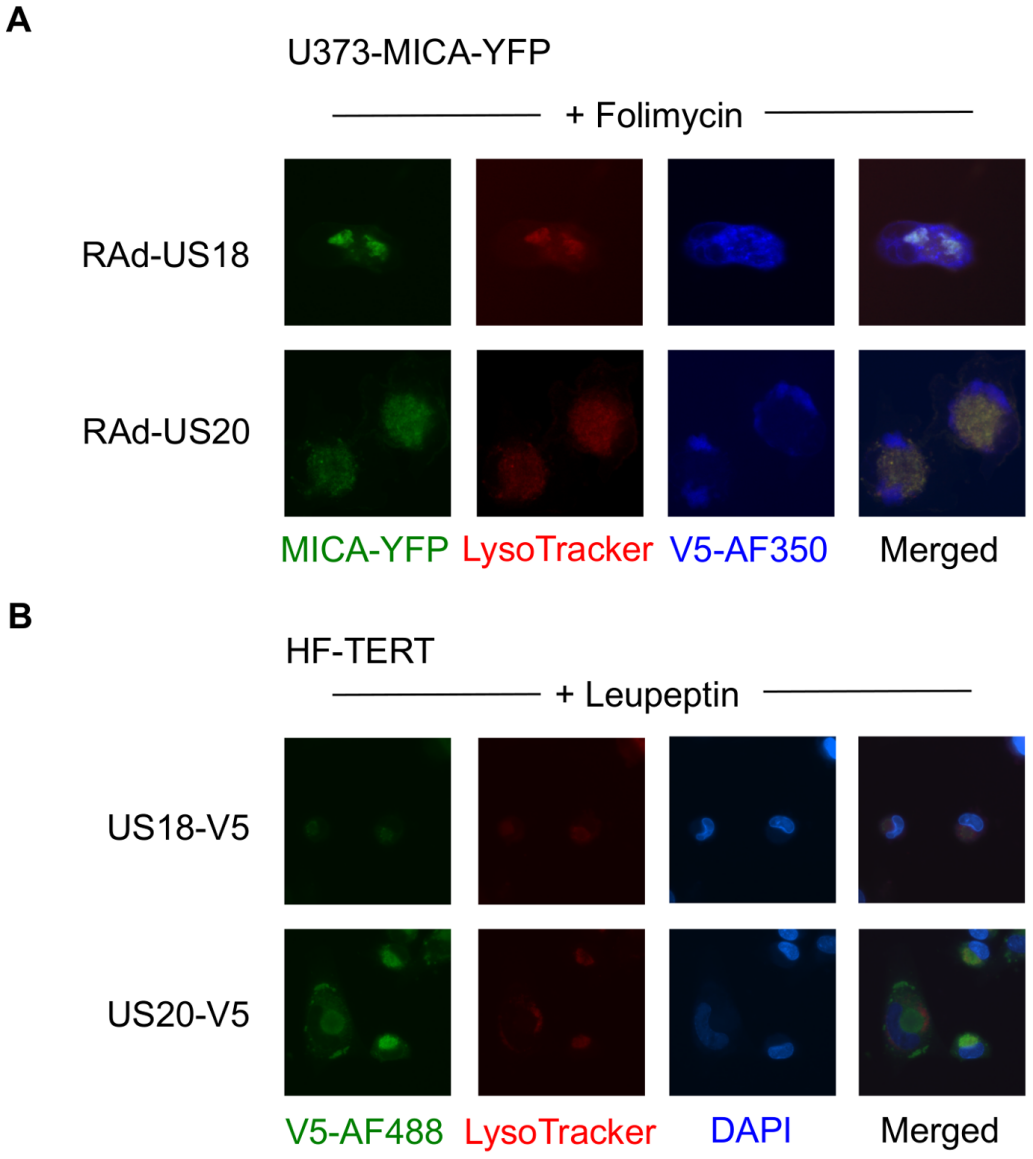
Targeting of MICA-YFP within lysosomes. A. U373 cells stably transfected with MICA-YFP were infected with US18- or US20 expressing adenovirus for 72 h. Folimycin was added 12 h prior to analysis. Cells were incubated with Lysotracker Red DND-99 (1∶1000) for 30 mins, washed once in DMEM and twice in PBS, fixed and permeabilized, and immunostained with a mouse antibody for the V5 epitope tag plus anti-mouse IgG-Alexa Fluor 350 secondary antibody. Results are representative of two independent experiments. B. HF-TERTs were infected with US18-V5 tagged HCMV or US20 V5-tagged HCMV for 72 hr. Leupeptin was added 12 h prior to analysis. Cells were incubated with Lysotracker Red DND-99 (1∶1000) for 30 mins, washed once in DMEM and twice in PBS, fixed and permeabilized, and immunostained with a rabbit antibody for the V5 epitope tag plus anti-rabbit IgG-Alexa Fluor 488 secondary antibody and counterstained with the nuclear dye DAPI. Results are representative of two independent experiments.

To examine further the effect of these genes on MICA, the genes were delivered to a U373 cell line expressing a MICA-yellow fluorescent protein (YFP) fusion protein (Fig. 6). MICA-YFP expression was suppressed efficiently by US18 or US20, but could be restored by treatment with the lysosomal inhibitor, folimycin (Fig. 6). In the presence of folimycin, US18 expression caused MICA-YFP to be redistributed to punctate intracytoplasmic structures, in which MICA-YFP was co-localized with the US18 protein (pUS18; Fig. 6). Although US20 expression also induced MICA-YFP to traffic to similar structures, the US20 protein (pUS20) did not localize to this compartment (Fig. 6). Expression of US19 as a control had no effect on MICA distribution (Fig. 6). To investigate whether these intracellular structures represented lysosomes, we performed similar experiments using a lysosomal staining reagent (Lysotracker Red DND-99) (Fig. 7A). These experiments showed that the MICA-YFP signal in both pUS18- and pUS20-expressing cells treated with folimycin co-localized with the lysosomal staining. The pUS18 staining was also lysosomal, whereas that of pUS20 was not. We also investigated whether pUS18 and pUS20 co-localized with the lysosomes in the context of HCMV infection (Fig. 7B). This analysis did not clearly reveal an association of pUS18 and pUS20 staining with that of lysosomes, although there was some co-localization in a proportion of cells with a strong cytopathic effect. In many cells, pUS20 appeared to be present in an intracellular compartment adjacent to the lysosomal staining. We concluded that both proteins promote proteolysis of MICA in the lysosome.

### US18 and US20 both encode NK cell evasion functions

**Figure 8:**
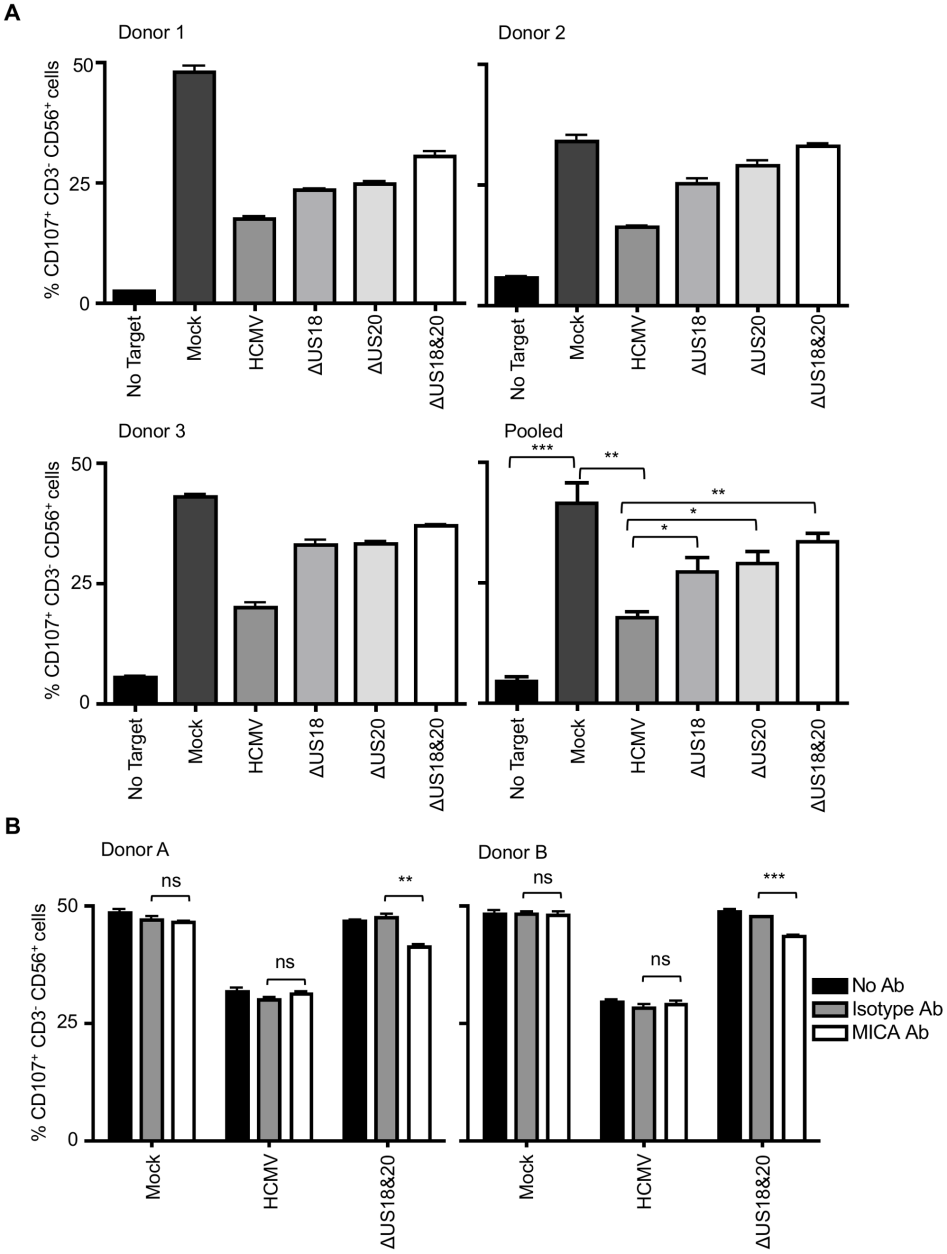
Loss of US18 and US20 increases NK degranulation in response to HCMV-infected targets. A. Fibroblasts (HF-TERTs) were mock infected or infected with the Merlin strain of HCMV or mutants lacking US18 (ΔUS18), US20 (ΔUS20) or combination of US18 and US20 (ΔUS18, ΔUS20) for 72 h. Infected cells were incubated with donor PBMC for 5 h and NK degranulation assessed by % CD107^+^ cells within the CD3^−^, CD56^+^ population by flow cytometry. Results are shown from 3 separate donors performed in triplicate and the mean of pooled results was analysed by Student's t-test (*P<0.05, **P<0.01, ***P<0.001). B. HF-TERTs were mock infected or infected with the Merlin strain of HCMV or the US18 and US20 deletion mutant for 72 h. Infected cells were incubated with isotype control or a MICA blocking antibody for 30 min prior to incubation with donor PBMC for 5 h. NK degranulation was assessed by % CD107^+^ cells within the CD3^−^, CD56^+^ population by flow cytometry. Results are shown from 2 separate donors performed in triplicate and the results were analysed by Student's t-test (**P<0.01, ***P<0.001, ns = not significant).

Before designating a virus gene as being an NK cell evasion function, it is important to monitor its biological activity during infection. The effects of US18 and US20 on NK cell recognition were therefore analyzed using the HCMV deletion mutants described above (Fig. 8A, Fig. S10). Relative to uninfected cells, infection with strain Merlin elicited robust protection against NK cells in all donors tested. A significant increase in NK cell degranulation was associated with loss of US18 or US20 (Fig. 8A) or the US18–22 ‘block’ deletion (Fig. S10B), and an additive effect was observed when both genes were absent. We concluded that US18 and US20 are effective in suppressing NK cell activation in the context of a productive HCMV infection. The use of a MICA blocking antibody led to a small decrease in NK degranulation in response to targets infected with the US18 and US20 double deletion virus, whilst the same antibody had no effect on NK activation in response to Mock or HCMV-infected targets (Fig. 8B). These data suggest that either the blockade of MICA was incomplete or that US18 and US20 target other cellular molecules capable of regulating NK cell activation.

## Discussion

HCMV-infected fibroblasts are extraordinarily resistant to NK cells as assessed *in vitro* by cytolysis or CD107-mobilization assays [29], [30]. Nonetheless, NK cells play a critical role in combating infections *in vivo*. We set out to determine whether the rapid up regulation of NKG2DL transcription driven by expression of the major HCMV IE genes would result in transient exposure of the activating ligands MICA, MICB, or ULBP2 early during infection [44], [45], thereby creating a window of opportunity for NK cell recognition. These studies revealed an interesting differential effect; HCMV IE1 preferentially unregulated the NKG2DL ULBP2, whereas IE2 predominantly activated MICA/B. Although IE1 and IE2 are encoded from within the same transcriptional unit by differential splicing, they are functionally distinct. IE1 is not essential for viral replication, but enhances replication by targeting intrinsic barriers to viral infection (PML-bodies, hDaxx, STAT-2, and p107), whereas IE2 is essential and is a potent transcriptional transactivator [55]. It is logical that individual NKG2DL should be differentially sensitive to specific triggers of cellular stress. Indeed, the dichotomy presented by the major HCMV IE genes provides a tractable experimental system by which to explore the cellular mechanisms that underpin NKG2DL regulation. Despite their up regulation by IE genes, HCMV was observed to suppress NKG2DL expression efficiently right through the early and late phases of the replication cycle.

Activation of NKG2DL expression during infection could only be detected when the viral genome had been engineered to delete relevant NK cell evasion functions; studies using such deletion mutants elegantly reveal the relative contributions made by individual NK cell evasion genes in mounting a comprehensive defense. While the control of MICB, ULBP1 and ULBP2 by UL16 was fully consistent with previous findings [46], unexpectedly the UL142 deletion mutant had no overt effect on MICA or ULBP3 expression. Strain AD169 controlled MICA expression despite containing a 15 kbp deletion that includes UL142 (this study; [56]). These observations prompted us to search for additional HCMV functions capable of targeting MICA, which were mapped to US18 and US20 using a combination of HCMV ‘block’ deletion mutants on a ΔUL16,ΔUL18,UL32-GFP background and adenovirus vectors expressing individual HCMV genes. Human viruses are known to eliminate host proteins selectively by recruiting cellular proteasomal or lysosomal proteolytic pathways. A specific role for the lysosome in the proteolytic degradation of MICA in HCMV-infected cells was identified.

The functions of US18 and US20 were validated in the context of HCMV productive infection. Deletion of US18 and US20 individually was associated with either no effect or a modest but significant increase in MICA cell surface expression relative to HCMV-infected cells, and both of these deletions enhanced NK cell activation in a functional assay. pUS18 and pUS20 are thus able individually to compensate for each other's loss in regulating MICA. Deletion of both genes together reduced the efficiency of MICA down regulation in a more than additive fashion, and yet consistently resulted in only a modest increase in NK cell activation above that observed for the single US18 and US20 deletions. One possible explanation of this is that the MICA antibodies used may not recognize all glycosylated isoforms of MICA with equal efficiency. Also, NK cells are controlled by thresholds, and thus cannot be expected to respond with linear kinetics to levels of MICA expression.

Neither US18 nor US20 has been assigned a function previously. However, both genes belong to the HCMV US12 family, which consists of ten tandemly arranged genes (US12–US21) encoding distantly related seven-transmembrane domain proteins [46]. A low level of amino acid sequence similarity has been noted between some US12 gene family members and the transmembrane BAX-inhibitor motif containing protein (TMBIM) superfamily [57], [58], [59]. The 6 TMBIM family proteins (TMBIM1–6) have functions relating to apoptosis and regulation of ER stress [60], and TMBIM6 (BAX-inhibitor 1) regulates lysosomal degradation of Gb3 and P450 2E1 [61], [62]. Although a poxvirus-encoded TMBIM4 homolog (viral GAAP, Golgi anti-apoptotic protein) has been shown to suppress apoptosis [63], no such function has yet been assigned to any US12 family member. The significance, if any, of the similarity of US12 family proteins to TMBIM family proteins in evolutionary and functional terms remains unknown. The same evaluation applies to the more marginal similarities to G protein-coupled receptors [53].

The HCMV genome contains 15 families of related genes, each potentially acquired via a process of gene duplication [51], [64]. The US12 family may have arisen as a genomic “accordion”, which involves the rapid expansion of a single gene under a strong selective pressure, to an array of related genes, which may collapse subsequently if the selective pressure wanes. This feature was recently demonstrated experimentally in a poxvirus (vaccinia virus) to facilitate selective adaption to the host's intrinsic antiviral defences [65]. Expansion of the US12 family is likely to have long pre-dated the speciation of humans, as the family is well conserved in cytomegaloviruses of chimpanzee and Old World primates (Rhesus and Cynomolgus macaques), which encode the same genomic arrangement of 10 recognizable US12 family genes. Several US12-related genes are also present in cytomegaloviruses of New World primates (Green Monkey and Owl Monkey), although these vary in number (11 and 7 genes respectively) [49], [51], [66], [67], [68], [69]. In support of a genomic accordion expansion, the ten US12 family members (US12–US21) are encoded tandemly in a gene cluster that is transcribed in three transcriptional cassettes: US12–US17, US18–US20, and US21 independently [70], [71], [72].

The presence of the US12 family in primate cytomegalovirus genomes implies that the selective pressure that initially induced the expansion has been maintained in some form, and the various numbers and arrangements of US12-related genes indicates that the family has continued to adapt and diverge in order to acquire an expanded functional range. Interestingly, the US6 gene family (US6–US11) contains multiple members (US6, US10, and US11) that target the MHC-I antigen-processing pathway, with the individual proteins targeting various parts of the pathway in order to provide greater effectiveness and resistance to mechanisms of host resistance [13], [14], [73], [74]. In this context, it is interesting to note that efficient down regulation of MICA requires both US18 and US20. Further functional studies may provide additional insights into the evolution and functions of the US12 family.

US18 and US20 now join with UL16, UL142 and miR-UL112 in expanding to five the set of HCMV genes that have been demonstrated to counter the action of the single NK cell activating receptor, NKG2D. In addition to its role in regulating the function of NK cells, NKG2D is also expressed ubiquitously on γδ-T cells and a subset of αβ-T cells. The number of HCMV-encoded gene products targeting the NKG2D pathway emphasizes the importance of this activating receptor in the immune response to HCMV.

## Materials and Methods

### Ethics statement

Healthy adult volunteers provided blood and dermal fibroblasts for this study following written informed consent (approved by the Cardiff University School of Medicine Ethics Committee Ref. no: 10/20).

### Cell lines

Human fetal foreskin fibroblasts immortalized by human telomerase (HF-TERT), HF-TERTs transfected with the Coxsackie-adenovirus receptor (HF-CAR), donor dermal fibroblasts (primary and TERT-immortalized), and U373 MICA-YFP-expressing cells (were maintained at 37°C in 5% CO_2_ in growth medium (Dulbecco's minimal essential medium (DMEM) supplemented with penicillin/streptomycin and 10% fetal calf serum (Invitrogen, Paisley, UK)) [75]. U373-MICA-YFP cells were constructed by transfecting full-length MICA allele (Genbank Accession no. AAD52060) fused to YFP, (a gift from Professor Dan Davis, University of Manchester, UK, generated as previously described [76]) into U373 cells with Effectene (Qiagen, Manchester, UK) and adding drug selection using 0.75 mg/ml G418 (Invitrogen).

### MICA typing

DNA was extracted from donor blood samples using the Qiagen Blood and tissue DNA extraction kit. MICA typing was performed by the Anthony Nolan Trust, as previously described [54].

### Viruses

HCMV deletion mutants were generated by recombineering of the bacterial artificial chromosome (BAC) of HCMV strain Merlin (GenBank accession number GU179001.1), as described previously [77]. Strain Merlin contains the complete genetic complement of HCMV, and is frame shifted in two genes (RL13^−^, UL128^−^). The regions encompassing the sites recombineered in BACs were verified by PCR and sequencing. A list of HCMV constructs, indicating deleted genes, other modifications, and the primers used in their production, is shown in Text S1. The HCMV strain AD169 (varUK; Genbank accession number BK000394) previously described was also used in some experiments [78], [79]. Recombinant adenoviruses (RAds) were generated as described previously [80]. Briefly, HCMV genes were amplified from the strain Merlin BAC by using primers containing arms of homology to the adenovirus BAC vector (pAL1141) and recombineered into pAL1141. RAd-IE1 and IE2 contained IE1 and IE2 from HCMV strain AD169 cloned into the adenovirus BAC vectors. A list of RAds used and the primers used to generate them is shown in Text S2.

### Viral infections

Cells were seeded in growth medium at appropriate cell densities (1×10^6^ cells for a 25 cm^2^ flask, 5×10^5^ cells per well for a 6-well plate, 5×10^4^ cells per well for a 24-well plate). The following day, the cells were infected with virus at the required multiplicity of infection in an appropriate volume of growth medium (2 ml for a 25 cm^2^ flask, 1 ml per well for a 6-well plate, 250 µl per well for a 24-well plate) for 2 h on a rocker at 37°C in 5% CO_2_. The inoculum was then replaced with fresh growth medium (7 ml for a 25 cm^2^ flask, 4 ml per well for a 6-well plate, 1 ml per well for a 24-well plates), and the cells were incubated for the required times. Fetal calf serum was omitted from the growth medium for HCMV infections. For inhibitor studies, cells were treated 12 h prior to harvesting with proteasomal (MG132 10 µM) or lysosomal inhibitors (folimycin 1 µM, leupeptin 200 µM or chloroquine 100 µM) in DMEM.

### Flow cytometric analysis of cell surface marker expression

Cells were harvested by washing once with phosphate-buffered saline (PBS) and treating with 1× trypsin/EDTA for 1 min at 37°C. After neutralizing the trypsin with growth medium, the cells were washed once in flow cytometry (FC) buffer (1% bovine serum albumin and 0.05% sodium azide in PBS). The cells were resuspended in an unconjugated primary antibody (murine IgG, Sigma Aldrich, Poole, UK, 1∶1000 dilution in FC buffer; anti-MICA clone AM01, anti-MICB clone BM02, anti-MICA/B clone BAM01, anti-ULBP2 clone BUM01, BAMOMAB GmBH, Graefelfing, Germany, 1∶400 dilutions in FC buffer; anti-ULBP1 Clone 170818, MAB1380, anti-ULBP3 Clone 166510, MAB1517, R&D Systems, Abingdon, UK, 1∶200 dilutions in FC buffer; anti-MHC-I, clone W632, AbD Serotec, Kidlington, UK, 1∶2000 dilution in FC buffer) and incubated for 30 min at 4°C. The cells were then washed twice with FC buffer and incubated in an Alexa Fluor 647 conjugated anti-mouse IgG secondary antibody (Invitrogen, 1∶500 dilution in FC buffer) for 30 min at 4°C. After 3 further washes in FC buffer, the cells were fixed in 2% paraformaldehyde (PFA) for 10 min and analyzed by using an Accuri Cflow cytometer (BD Biosciences, Oxford, UK). A forward scatter and side scatter dot plot was used to gate both on viable cells and infected cells (Fig. S1A). The median fluorescence intensity (MFI) was used in subsequent analysis.

### Triton X-114 extraction of membrane proteins

Cells were washed once with ice-cold PBS, scraped into 4 ml ice-cold PBS, recovered by centrifugation (1,600 rpm for 3 min), and stored at −20°C. They were then resuspended in 300 µl Triton X-114 extraction buffer (2% Triton X-114, 1∶100 protease inhibitors, and 1 mM DTT in PBS) and sonicated. The lysate was clarified by microcentrifugation at 13,000 rpm for 1 h at 4°C in a 1.5 ml non-stick tube. The supernatant was transferred to a fresh 1.5 ml non-stick tube, incubated at 37°C for 10 min, and microcentrifuged at 13,000 rpm for 5 min at 37°C. The upper phase was removed, leaving a bead of Triton-X114 of approximately 60 µl. PBS was added up to a total volume of 300 µl and the samples sonicated. The extracted protein was precipitated by adding 1.2 ml precipitation reagent (Merck protein precipitation kit 539180) and incubating at −20°C for 1 h, and collected by centrifuging at 13,000 rpm for 10 min at 4°C and aspirating the supernatant. The precipitated proteins were washed once with 500 µl wash buffer (Merck protein precipitation kit 539180) and collected by centrifuging at 13,000 rpm for 10 min at room temperature and aspirating the supernatant. The pellets were allowed to air dry for a few minutes and then resuspended either in 1× Nupage gel sample buffer (Invitrogen) plus 10 mM DTT for direct immunoblotting or in 1× denaturing buffer (NEB) for EndoH and PNGase F treatment. Samples were denatured at 95°C for 10 mins or for analysis of US18 and US20 expression at 50°C for 10 mins. Denaturation at 95°C was found to lead to high molecular weight smears of US18 and US20 in resulting immunoblots, presumably due to formation of aggregates as noted for other polytopic membrane proteins.

### Immunoblotting

Protein samples (20 µl) were separated on ready-made 10% Nu-PAGE polyacrylamide gels (Invitrogen) by SDS-PAGE and transferred to Hybond-P nitrocellulose (GE Life Science) by semi-dry blotting. Nitrocellulose membranes were prepared by treating with 5 ml Pierce MISER antibody extender (Fisher Scientific) for 10 min and washing 7 times with distilled water. They were then blocked in 5% milk in Tris-buffered saline containing 0.05% TWEEN-20 and 0.05% Triton X-100 (TBS-T-T) overnight at 4°C. The membranes were then incubated with primary antibodies diluted in 5% milk in TBS-T-T overnight at 4°C. They were then washed 5 times for 5 min in TBS-T-T, and incubated in anti-mouse IgG-HRP conjugate (Insight Biotechnology Ltd, Wembley, UK, 1∶5,000–1∶10,000) for 1 h at room temperature. After washing a further 5 times for 5 min in TBS-T-T, the membranes were incubated for 5 min in SuperSignal West Pico Chemiluminescent substrate (Fisher Scientific) before being exposed to Hyperfilm-MP film (GE Life Science, Little Chalfont, UK) for development. The blots were stripped in Pierce Stripper buffer (Fisher Scientific) for 10 min, washed 7 times in TBS-T-T, reblocked, and reprobed.

### Immunofluorescence staining and analysis

Cells were grown and infected with virus in glass-bottomed 24-well plates. At various times p.i., the cells were washed with PBS, and fixed with 2% PFA for 10 min. In some instances, cells were incubated with LysoTracker Red DND-99 (Cat. no. L7528, 1∶1000 in complete DMEM, Invitrogen) for 30 min, washed once with complete DMEM, then PBS and and fixed with 2% PFA for 10 min. The fixed cells were washed twice with IC buffer (0.2% saponin, 1% BSA, 0.05% sodium azide in PBS) and incubated with primary antibodies (anti V5-tag, AbD Serotec) diluted in IC buffer for 1 h at 4°C. The cells were then washed 3 times with IC buffer and incubated with Alexa Fluor 594 or 350 (Lysotracker experiments)-conjugated anti-mouse IgG antibodies (Invitrogen) for 1 h at 4°C. The cells were washed once with IC buffer and where required incubated for 10 min with DAPI (0.5 µg/ml) diluted in IC buffer. They were then washed twice further in IC buffer, before the addition of 2% PFA and analysis by fluorescent microscopy.

### Intracellular flow cytometry

Cells were harvested by washing once with phosphate-buffered saline (PBS) and treating with 1× trypsin/EDTA for 1 min at 37°C. After neutralizing the trypsin with growth medium, the cells were washed with PBS and fixed with 2% PFA for 10 mins. The fixed cells were washed twice with IC buffer and incubated with anti-V5 tag antibody (1∶2000) or control mouse IgG diluted in IC buffer for 1 hr at 4°C. The cells were washed twice with IC buffer and incubated with Alexa Fluor 647-conjugated anti-mouse IgG antibodies (1∶500) for 30 mins at 4°C. They were then washed three times in IC buffer, before the addition of 2% PFA and analysis by flow cytometry.

### NK cytotoxicity assays

NK cytotoxicity was assessed by standard ^51^Cr chromium release assay as described previously [39], using labelled RAd-infected fibroblast target cells. The effector∶target (E∶T) ratio was adjusted by using the number of NK cells present in the CD3^+^-depleted IFN-α-activated peripheral blood mononuclear cells (PBMC) used as effectors. Specific lysis (%) was calculated as [(experimental mean release - spontaneous mean release)/(maximum mean release - spontaneous mean release)]×100. The mean and SEM were determined from the results of triplicate or quadruplicate samples.

### NK degranulation assays

NK degranulation assays were performed in a similar manner to that described previously [39], [81]. Briefly, PBMC (approved by the Cardiff University School of Medicine Ethics Committee Ref. no: 10/20) and incubated overnight with IFN-α (1000 IU/ml) and IL-15 (15 ng/ml, Milenyi Biotech). PBMC (0.5–1×10^6^) were incubated for 6 h with 0.5–1×10^5^ fibroblast targets per well in a 96 well plate at an effector∶target (E∶T) ratio of 10∶1, with the addition of 3 µl per well FITC-conjugated anti-CD107 antibody (cat. no. 555800, clone H4A3, BD Biosciences) or 3 µl per well FITC-conjugated isotype control (cat. no. 555748, BD Biosciences), adding 1 µl/well BD GolgiStop (BD Biosciences) 1 h after beginning the incubation. (For antibody blocking experiments, targets were pre-incubated with anti-MICA (Clone 159277, mouse IgG2B, MAB1300, R&D Systems) or isotype control (MICB non-blocking antibody, Clone 236511, mouse IgG2B, MAB1599, R&D Systems) antibodies at a concentration of 10 µg/ml for 30 min prior to incubation with PBMC). PBMC were harvested and stained with conjugated antibodies against CD3 (anti-CD3 PE-Cy7, cat. no. 737657, Beckman Coulter, High Wycombe, UK) and CD56 (anti-CD56 PE, cat. no. A07788, Beckman Coulter), and fixed in 2% PFA before analysis by flow cytometry (BD Biosciences Accuri C Flow) (Fig. S10A).

## Supporting Information

Figure S1 **Regulation of NKG2DL expression during HCMV infection.** A. Fibroblasts (HF-TERTs) were mock-infected or infected with HCMV strain Merlin or deletion mutants of this strain lacking UL16 (ΔUL16) or UL142 (ΔUL142). Representative flow cytometry plots show the FSC/SSC gating strategy applied to gate on mock- and HCMV-infected cells. B. Fibroblasts (HF-TERTs) were mock-infected or infected with HCMV strain Merlin or deletion mutants of this strain lacking UL16 (ΔUL16) or UL142 (ΔUL142). Cell surface expression of MICA, MICB, MICA/B, ULBP2, MHC-I, or murine immunoglobulin (mIgG) was analyzed between 6 and 120 h p.i. by flow cytometry. Flow cytometry plots representative of two independent experiments are shown.
(TIFF)

Figure S2 **Regulation of ULBP1 and ULBP3 expression by HCMV expression.** A. Fibroblasts (HF-TERTs) were mock-infected or infected with HCMV strain Merlin or deletion mutants of this strain lacking UL16 (ΔUL16) or UL142 (ΔUL142). Cell surface expression of ULBP1, ULBP3, MHC-I, or murine immunoglobulin (mIgG) was analyzed between 6 and 120 h p.i. by flow cytometry. The results are shown as median fluorescence intensity (MFI) and are representative of 2 independent experiments. There was a small increase in ULBP3 on ΔUL16-infected cells. B. Flow cytometry plots representative of two independent experiments are shown.
(TIFF)

Figure S3 **HCMV strain AD169 down regulates MICA.** A. HF-TERTs were infected with HCMV strain AD169 at an m.o.i. of 10 for 72 h and MICA, MICB, MICA/B, ULBP2, and MHC-I expression were assessed by flow cytometry relative to a murine immunoglobulin (mIgG) control. The results are shown from three independent experiments. B. HF-TERTs were infected with HCMV strain AD169 at an m.o.i. of 10 for 72 h and MICA, MICB, MICA/B, ULBP2, and MHC-I expression were assessed by flow cytometry relative to a murine immunoglobulin (mIgG) control. Flow cytometry plots representative of 3 independent experiments are shown.
(TIFF)

Figure S4 **Regulation of NKG2DL by HCMV ‘block’ deletion mutants.** A. A schematic indicating the 10 HCMV ‘block’ deletions on the HCMV Merlin genome is shown. B. Fibroblasts (HF-TERTs) were mock infected or infected with HCMV or a deletion mutant lacking US18–22 (ΔUS18–22) for 72 h. Cell surface expression was analyzed by immunostaining and flow cytometry. Flow cytometry plots representative of 3 independent experiments are shown.
(TIFF)

Figure S5 **Detection of US18–22 V5 expression by intracellular flow cytometry.** Fibroblasts (HF-CARs) were infected with recombinant adenovirus expressing the individual US18–US22 genes for 72 h. V5 expression was analyzed by immunostaining with anti-V5 antibody or control mIgG and anti-mouse AF647 –conjugated secondary, followed by flow cytometry. Results are representative of three independent experiments.
(TIFF)

Figure S6 **Effect of US18–US22 expression in HF-CAR on cell surface expression of MICA.** Fibroblasts (HF-CARs) were infected with recombinant adenovirus expressing the individual US18–US22 genes for 72 h. MICA expression was analyzed by immunostaining with a MICA-specific antibody and anti-mouse AF647 conjugated secondary, followed by flow cytometry. A. The median fluorescence intensity (MFI) values from three independent experiments are shown. B. Representative flow cytometry plots are shown.
(TIFF)

Figure S7 **Analysis of MICA cell surface expression in cells infected with HCMV US18 and US20 deletion mutants.** A. Fibroblasts (HF-TERTs) were mock infected or infected with HCMV, ΔUS18 or ΔUS18, ΔUS20. Cell surface expression was analyzed by immunostaining and flow cytometry between 6 and 120 h p.i. Results shown are representative of two independent experiments. B. Fibroblasts (HF-TERTs) were mock infected or infected with HCMV, ΔUS18 or ΔUS18, ΔUS20 for 72 h. Cell surface expression was analyzed by immunostaining and flow cytometry. Flow cytometry plots representative of four independent experiments are shown.
(TIFF)

Figure S8 **Effect of US18 and US20 on different MICA alleles.** Dermal fibroblasts expressing different MICA alleles were mock infected or infected with HCMV, ΔUS18 or ΔUS18, ΔUS20 for 72 h. Cell surface expression was analyzed by immunostaining and flow cytometry. Flow cytometry plots are representative of three independent experiments.
(TIFF)

Figure S9 **Effect of proteasomal and lysosomal inhibitors on cell surface MICA/B expression in HCMV infection.** A. Fibroblasts (HF-TERTs) were mock-infected or infected with HCMV for 72 h. A proteasomal inhibitor (MG132) or lysososomal inhibitor (folimycin) was added 12 h prior to harvesting. Cell surface expression was analyzed by immunostaining and flow cytometry. The flow cytometry plots shown are representative of 3 independent experiments. B. Fibroblasts (HF-TERTs) were mock-infected or infected with HCMV for 72 h. The lysosomal inhibitors leupeptin or chloroquine were added 12 h prior to harvesting. Cell surface expression was analyzed by immunostaining and flow cytometry. The flow cytometry plots are representative of 2 independent experiments.
(TIFF)

Figure S10 **Analysis of NK cell activation by CD107 mobilization.** A. Representative flow cytometry plots show the gating strategy used for the CD107 mobilzation assays. Viable PBMCs were gated initially on a FSC/SSC gate and then the CD3− (PE-Cy7/FL4), CD56+ (PE/FL2) population was gated to allow analysis of CD107 staining (FITC/FL1). B. Mock-infected cells or cells infected with HCMV, parent (UL16^−^, UL18^−^, UL32-GFP), or ΔUS18–22 (UL16^−^, UL18^−^, US18–22^−^, UL32-GFP) virus for 72 hrs were used as targets in a CD107 mobilszation assay using blood bag-derived PBMC.
(TIFF)

Text S1 **HCMV constructs utilized and primers used in their generation.** A table shows the relevant HCMV viruses as they are referred to in the text, an internal (lab.) BAC reference and any genetic modifications they contain relative to the clinical HCMV Merlin strain. Primer sequences used in their generation are listed, which were used either to target selection cassettes or delete/insert sequences during recombineering reactions or for subsequent sequencing.
(DOCX)

Text S2 **Adenovirus constructs utilized and primers used in their generation.** A table shows the relevant Adenovirus constructs as they are referred to in the text, an internal (lab.) BAC reference and the gene insert. Primer sequences used in their generation are listed, which were used either to insert the gene sequence during recombineering reactions or for subsequent sequencing.
(DOCX)
