# Supplementary figures and images for: Two Novel Human Cytomegalovirus NK Cell Evasion Functions Target MICA for Lysosomal Degradation
Source: PLoS Pathog. 2014 May 1;10(5):e1004058. doi: 10.1371/journal.ppat.1004058 (PMC4006889; doi:10.1371/journal.ppat.1004058)

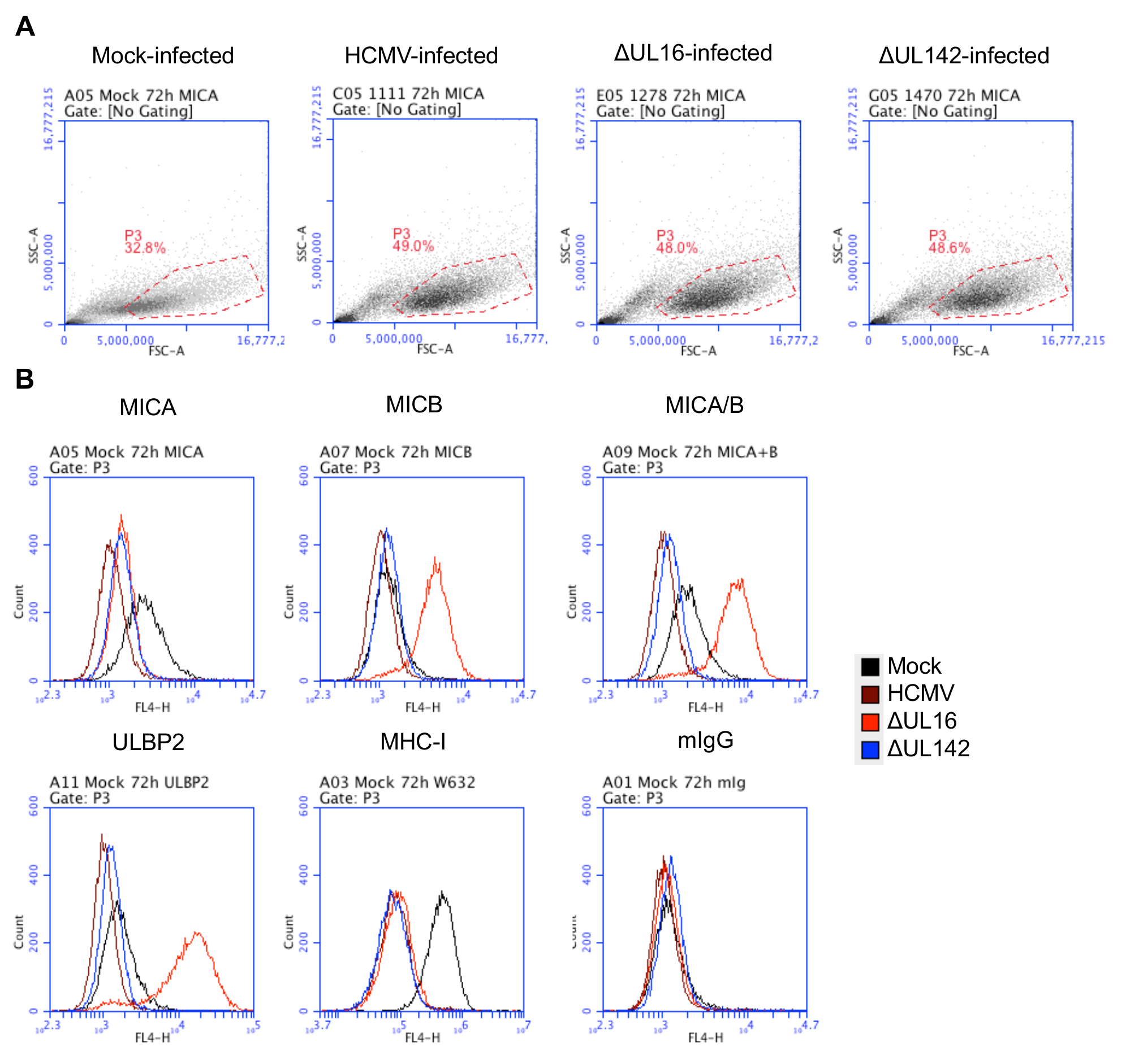

Supplement: Figure S1 — Regulation of NKG2DL expression during HCMV infection. A. Fibroblasts (HF-TERTs) were mock-infected or infected with HCMV strain Merlin or deletion mutants of this strain lacking UL16 (ΔUL16) or UL142 (ΔUL142). Representative flow cytometry plots show the FSC/SSC gating strategy applied to gate on mock- and HCMV-infected cells. B. Fibroblasts (HF-TERTs) were mock-infected or infected with HCMV strain Merlin or deletion mutants of this strain lacking UL16 (ΔUL16) or UL142 (ΔUL142). Cell surface expression of MICA, MICB, MICA/B, ULBP2, MHC-I, or murine immunoglobulin (mIgG) was analyzed between 6 and 120 h p.i. by flow cytometry. Flow cytometry plots representative of two independent experiments are shown. (TIFF) [file ppat.1004058.s001.tiff]

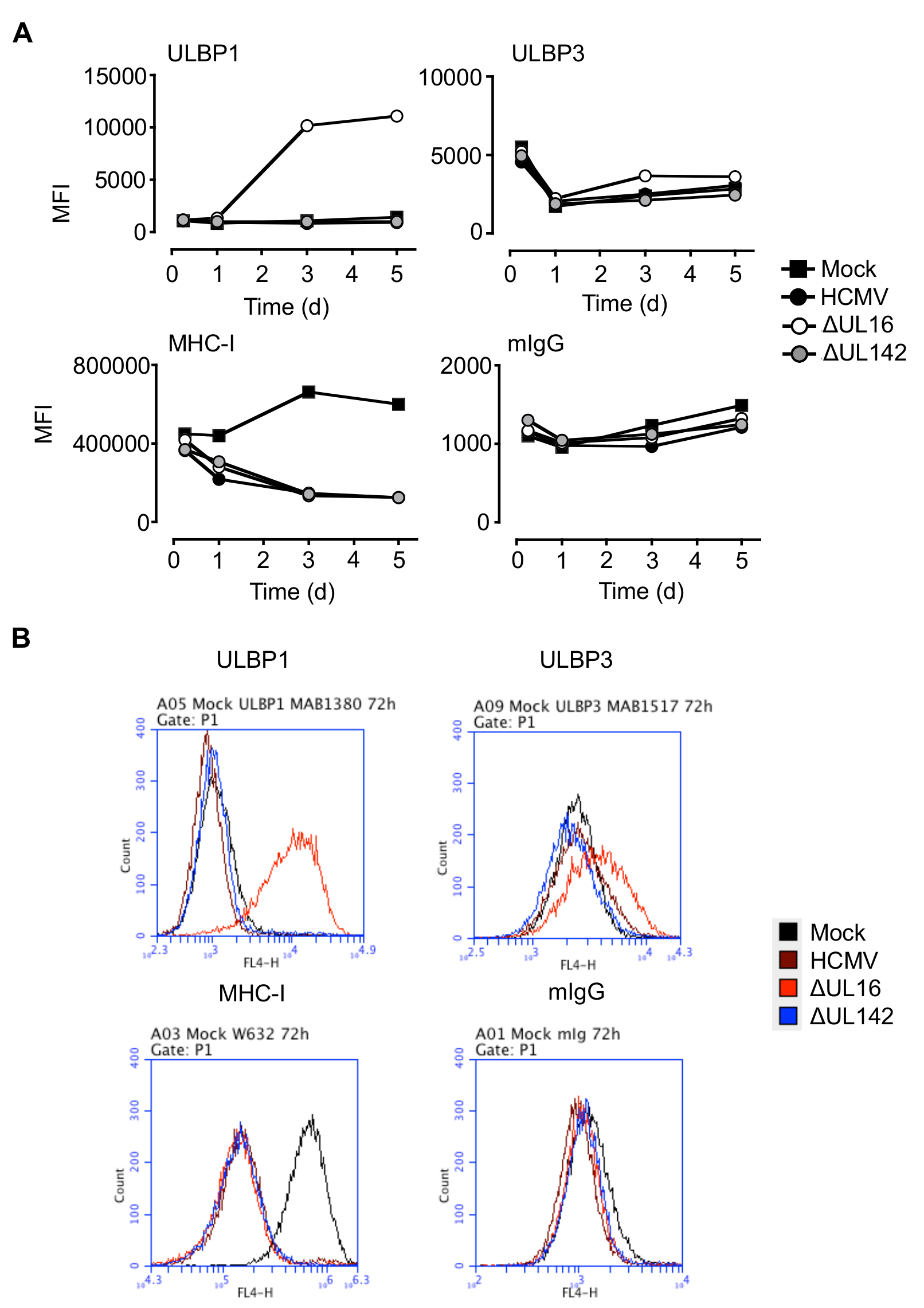

Supplement: Figure S2 — Regulation of ULBP1 and ULBP3 expression by HCMV expression. A. Fibroblasts (HF-TERTs) were mock-infected or infected with HCMV strain Merlin or deletion mutants of this strain lacking UL16 (ΔUL16) or UL142 (ΔUL142). Cell surface expression of ULBP1, ULBP3, MHC-I, or murine immunoglobulin (mIgG) was analyzed between 6 and 120 h p.i. by flow cytometry. The results are shown as median fluorescence intensity (MFI) and are representative of 2 independent experiments. There was a small increase in ULBP3 on ΔUL16-infected cells. B. Flow cytometry plots representative of two independent experiments are shown. (TIFF) [file ppat.1004058.s002.tiff]

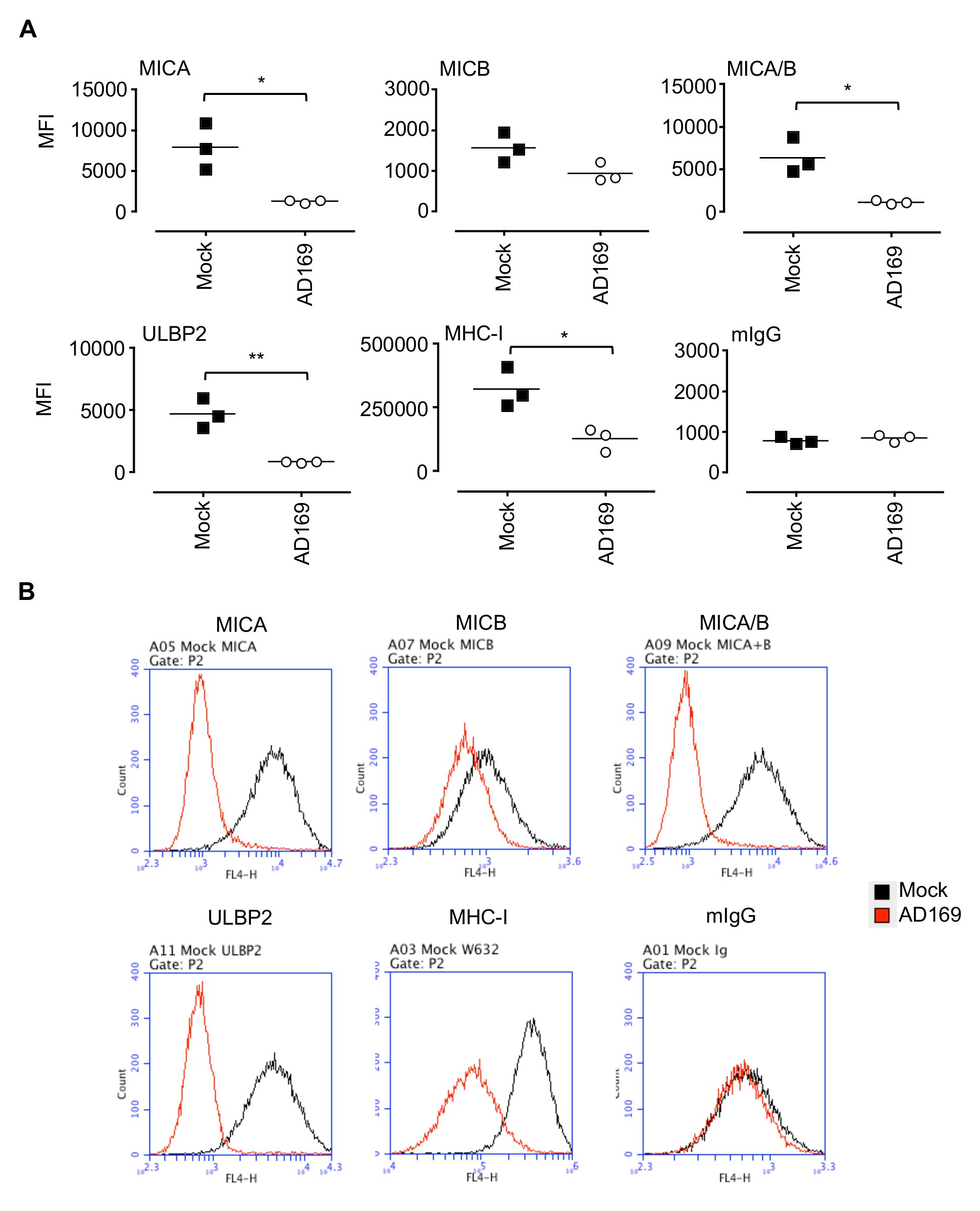

Supplement: Figure S3 — HCMV strain AD169 down regulates MICA. A. HF-TERTs were infected with HCMV strain AD169 at an m.o.i. of 10 for 72 h and MICA, MICB, MICA/B, ULBP2, and MHC-I expression were assessed by flow cytometry relative to a murine immunoglobulin (mIgG) control. The results are shown from three independent experiments. B. HF-TERTs were infected with HCMV strain AD169 at an m.o.i. of 10 for 72 h and MICA, MICB, MICA/B, ULBP2, and MHC-I expression were assessed by flow cytometry relative to a murine immunoglobulin (mIgG) control. Flow cytometry plots representative of 3 independent experiments are shown. (TIFF) [file ppat.1004058.s003.tiff]

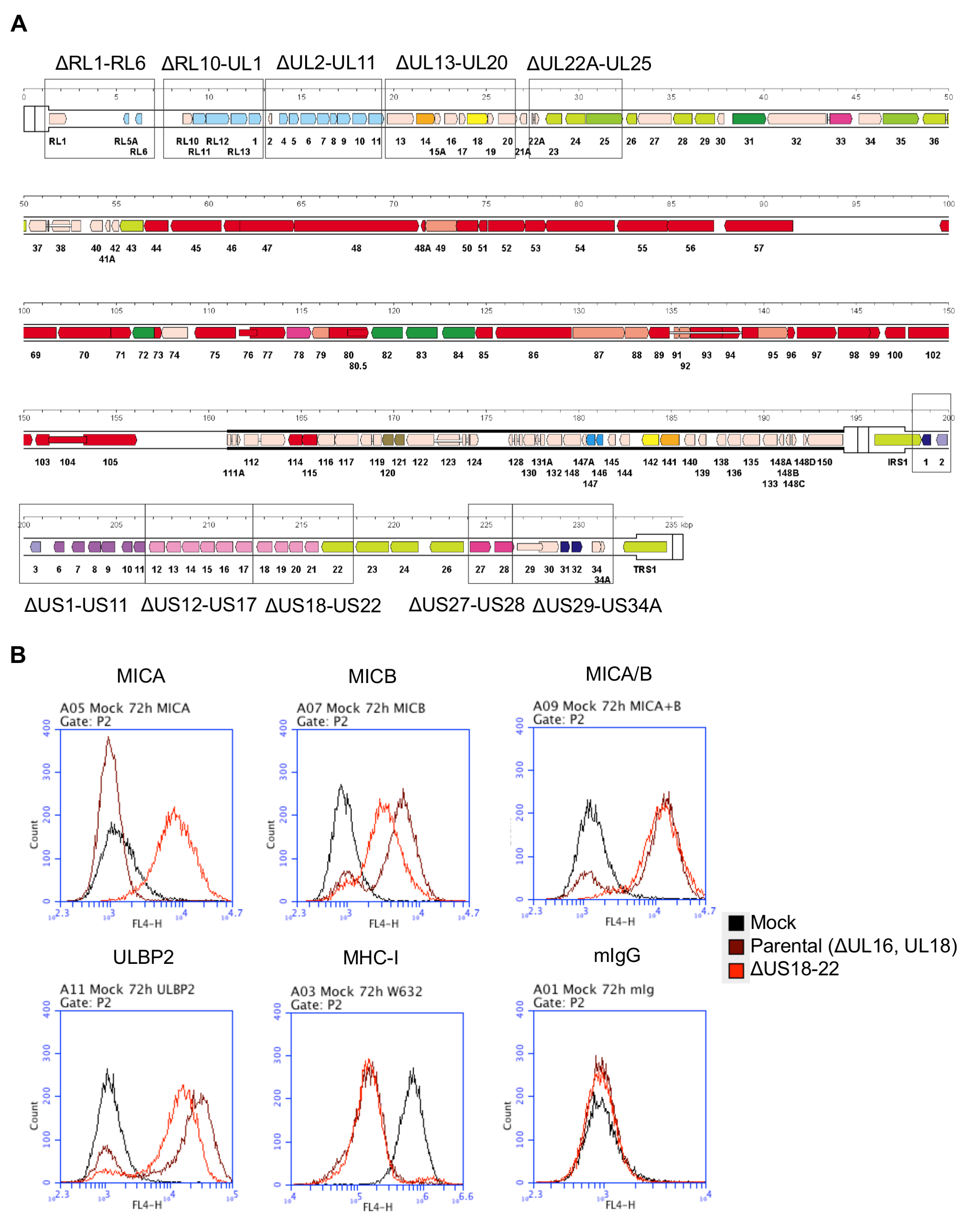

Supplement: Figure S4 — Regulation of NKG2DL by HCMV ‘block’ deletion mutants. A. A schematic indicating the 10 HCMV ‘block’ deletions on the HCMV Merlin genome is shown. B. Fibroblasts (HF-TERTs) were mock infected or infected with HCMV or a deletion mutant lacking US18–22 (ΔUS18–22) for 72 h. Cell surface expression was analyzed by immunostaining and flow cytometry. Flow cytometry plots representative of 3 independent experiments are shown. (TIFF) [file ppat.1004058.s004.tiff]

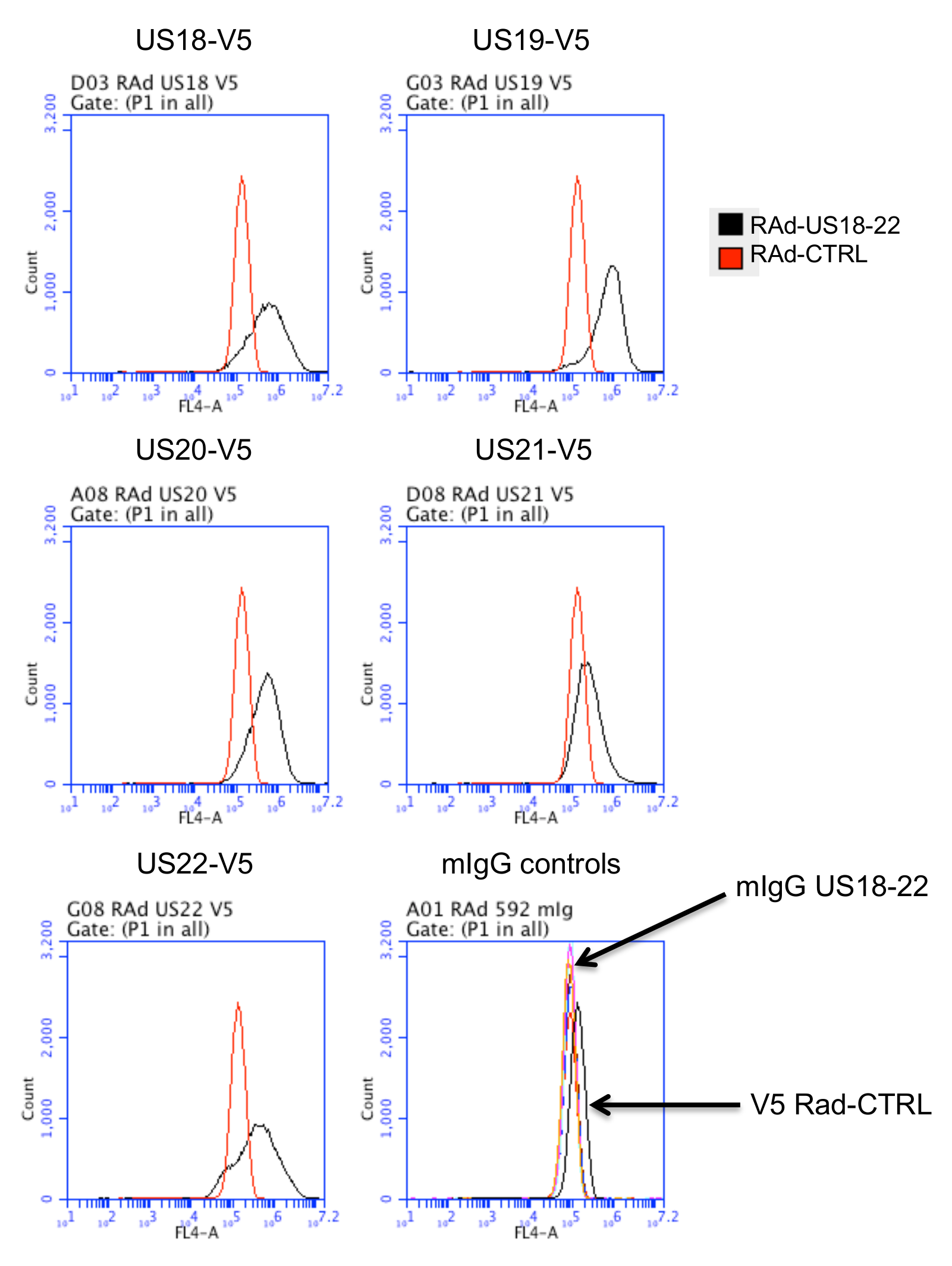

Supplement: Figure S5 — Detection of US18–22 V5 expression by intracellular flow cytometry. Fibroblasts (HF-CARs) were infected with recombinant adenovirus expressing the individual US18–US22 genes for 72 h. V5 expression was analyzed by immunostaining with anti-V5 antibody or control mIgG and anti-mouse AF647 –conjugated secondary, followed by flow cytometry. Results are representative of three independent experiments. (TIFF) [file ppat.1004058.s005.tiff]

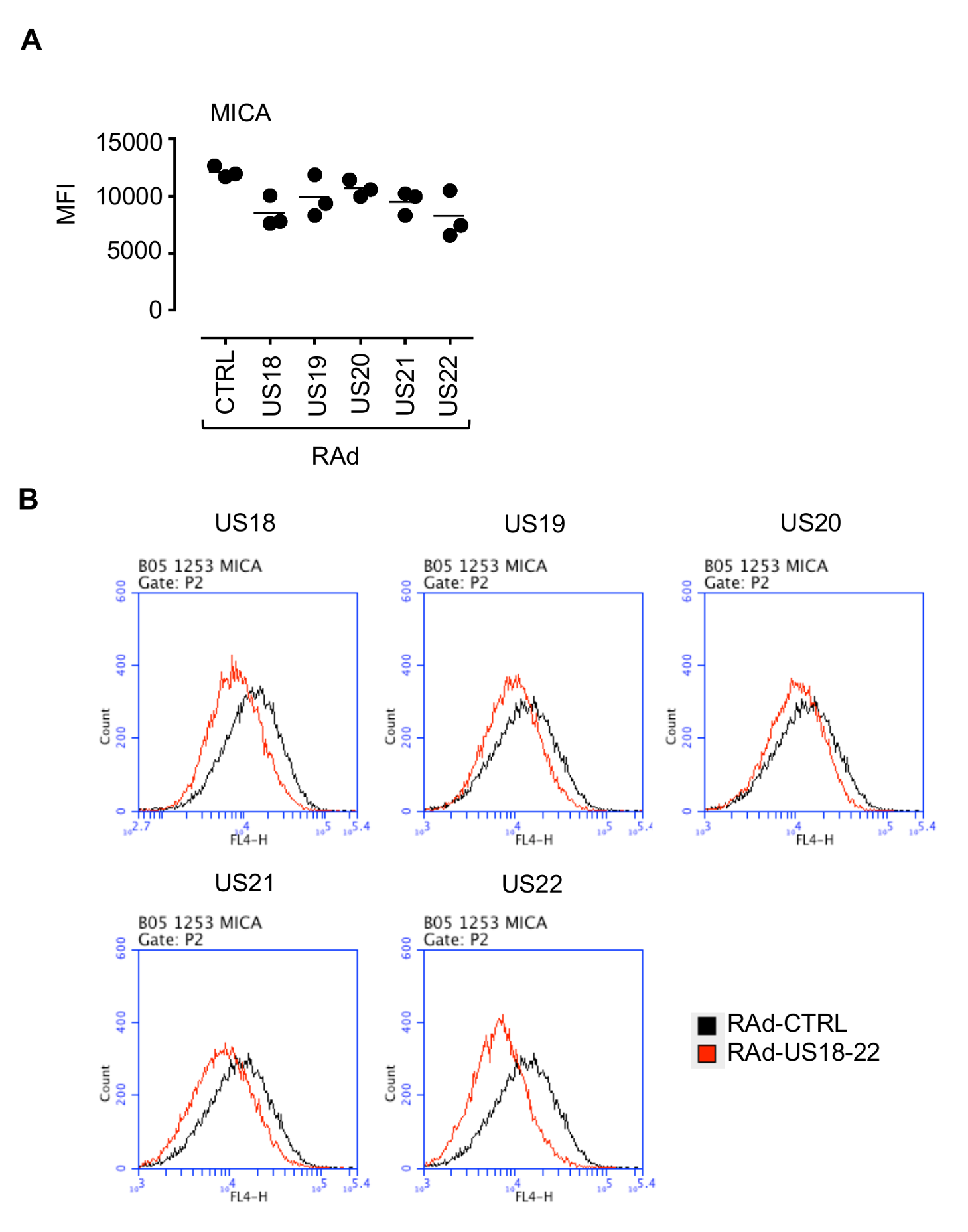

Supplement: Figure S6 — Effect of US18–US22 expression in HF-CAR on cell surface expression of MICA. Fibroblasts (HF-CARs) were infected with recombinant adenovirus expressing the individual US18–US22 genes for 72 h. MICA expression was analyzed by immunostaining with a MICA-specific antibody and anti-mouse AF647 conjugated secondary, followed by flow cytometry. A. The median fluorescence intensity (MFI) values from three independent experiments are shown. B. Representative flow cytometry plots are shown. (TIFF) [file ppat.1004058.s006.tiff]

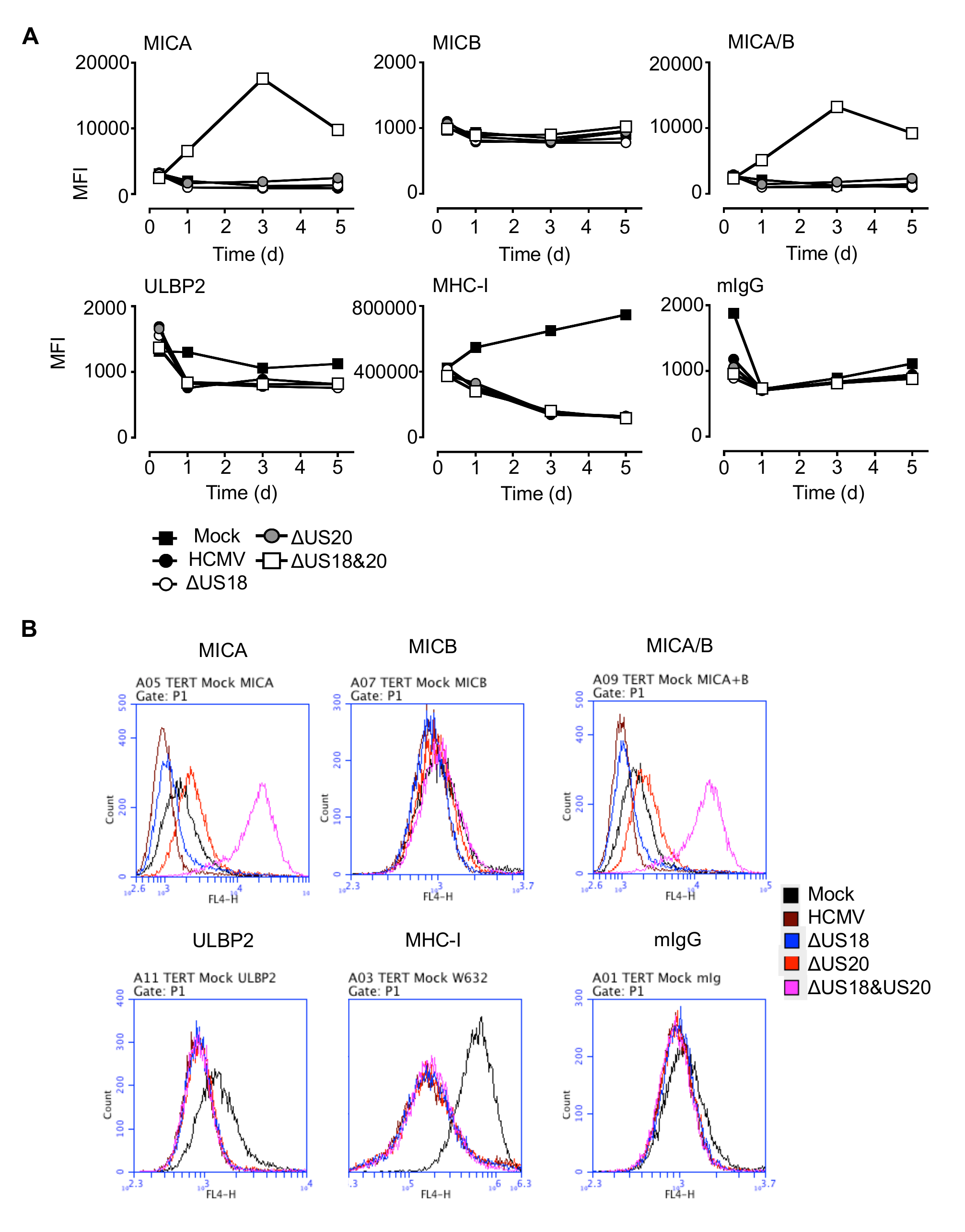

Supplement: Figure S7 — Analysis of MICA cell surface expression in cells infected with HCMV US18 and US20 deletion mutants. A. Fibroblasts (HF-TERTs) were mock infected or infected with HCMV, ΔUS18 or ΔUS18, ΔUS20. Cell surface expression was analyzed by immunostaining and flow cytometry between 6 and 120 h p.i. Results shown are representative of two independent experiments. B. Fibroblasts (HF-TERTs) were mock infected or infected with HCMV, ΔUS18 or ΔUS18, ΔUS20 for 72 h. Cell surface expression was analyzed by immunostaining and flow cytometry. Flow cytometry plots representative of four independent experiments are shown. (TIFF) [file ppat.1004058.s007.tiff]

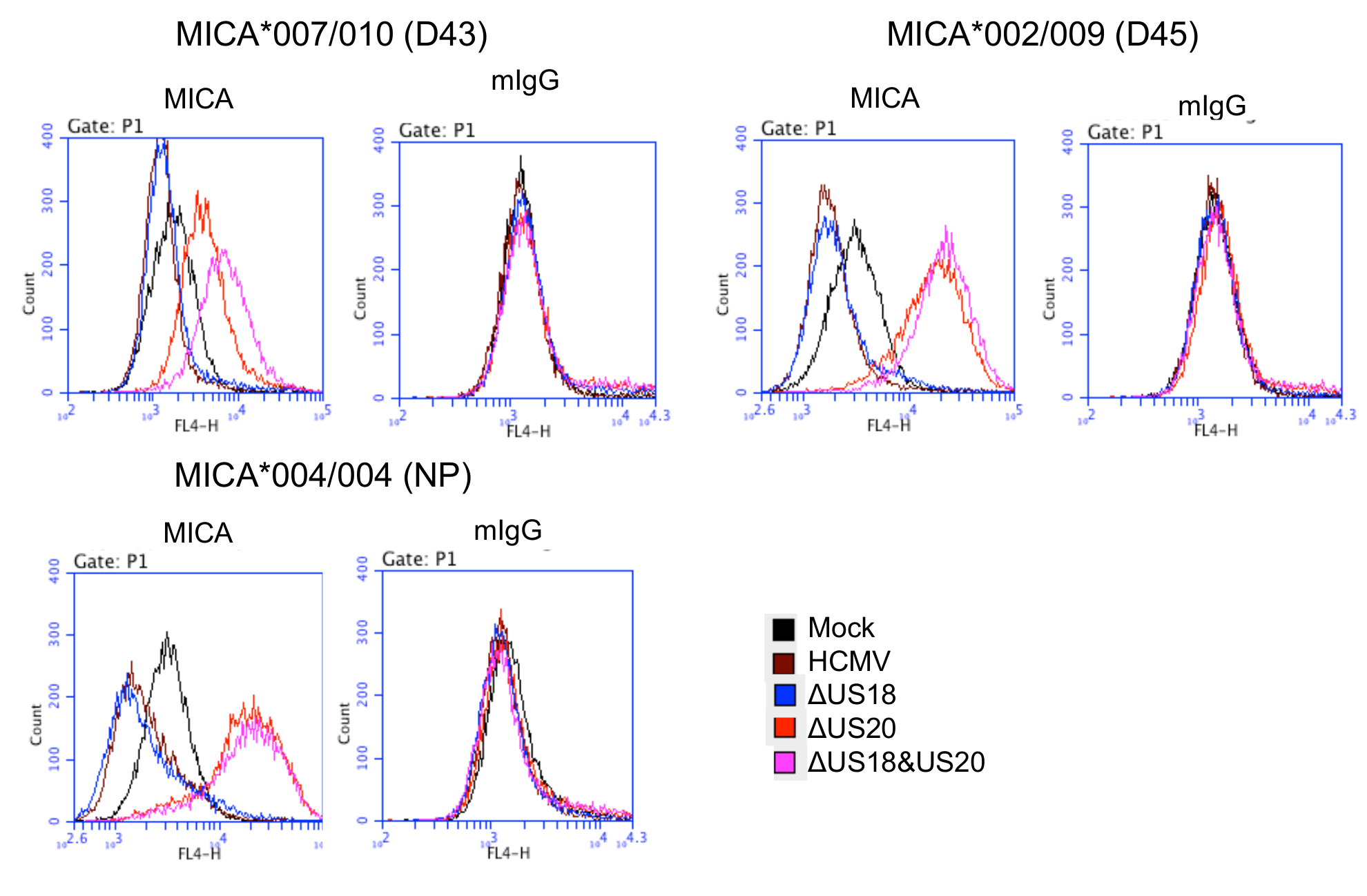

Supplement: Figure S8 — Effect of US18 and US20 on different MICA alleles. Dermal fibroblasts expressing different MICA alleles were mock infected or infected with HCMV, ΔUS18 or ΔUS18, ΔUS20 for 72 h. Cell surface expression was analyzed by immunostaining and flow cytometry. Flow cytometry plots are representative of three independent experiments. (TIFF) [file ppat.1004058.s008.tiff]

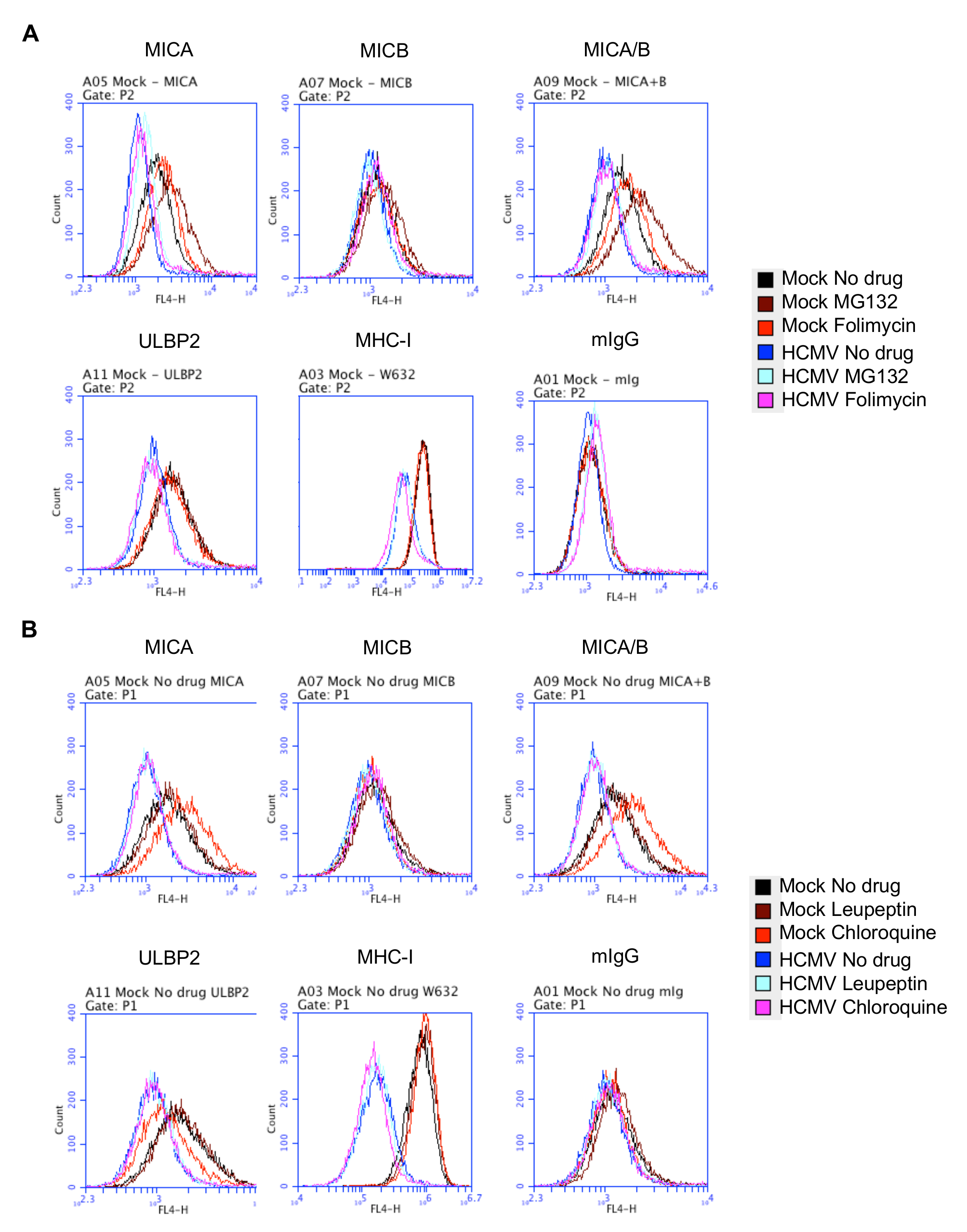

Supplement: Figure S9 — Effect of proteasomal and lysosomal inhibitors on cell surface MICA/B expression in HCMV infection. A. Fibroblasts (HF-TERTs) were mock-infected or infected with HCMV for 72 h. A proteasomal inhibitor (MG132) or lysososomal inhibitor (folimycin) was added 12 h prior to harvesting. Cell surface expression was analyzed by immunostaining and flow cytometry. The flow cytometry plots shown are representative of 3 independent experiments. B. Fibroblasts (HF-TERTs) were mock-infected or infected with HCMV for 72 h. The lysosomal inhibitors leupeptin or chloroquine were added 12 h prior to harvesting. Cell surface expression was analyzed by immunostaining and flow cytometry. The flow cytometry plots are representative of 2 independent experiments. (TIFF) [file ppat.1004058.s009.tiff]

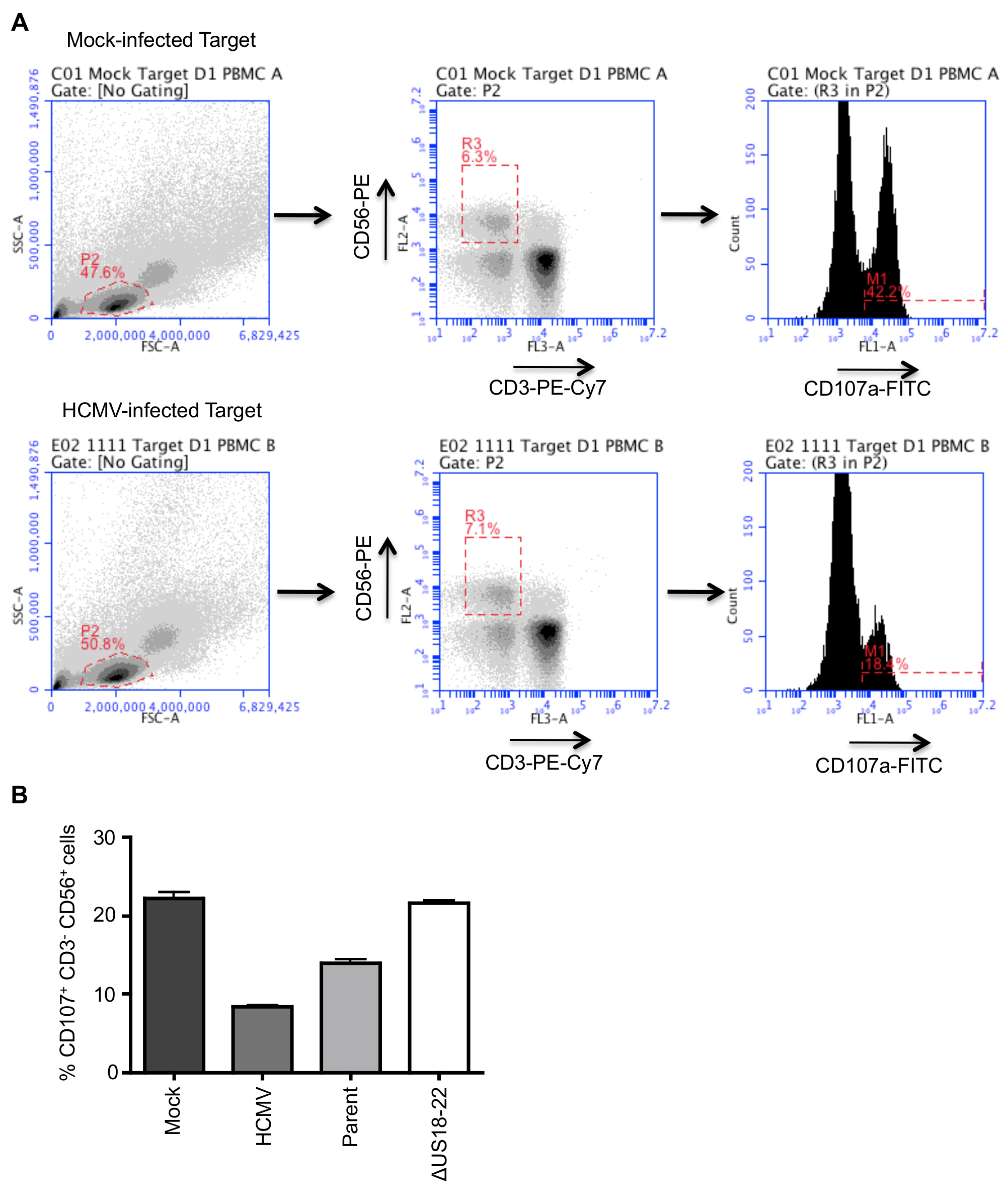

Supplement: Figure S10 — Analysis of NK cell activation by CD107 mobilization. A. Representative flow cytometry plots show the gating strategy used for the CD107 mobilzation assays. Viable PBMCs were gated initially on a FSC/SSC gate and then the CD3− (PE-Cy7/FL4), CD56+ (PE/FL2) population was gated to allow analysis of CD107 staining (FITC/FL1). B. Mock-infected cells or cells infected with HCMV, parent (UL16−, UL18−, UL32-GFP), or ΔUS18–22 (UL16−, UL18−, US18–22−, UL32-GFP) virus for 72 hrs were used as targets in a CD107 mobilszation assay using blood bag-derived PBMC. (TIFF) [file ppat.1004058.s010.tiff]
