# Supplementary material for: Two Novel Human Cytomegalovirus NK Cell Evasion Functions Target MICA for Lysosomal Degradation
Source: PLoS Pathog. 2014 May 1;10(5):e1004058. doi: 10.1371/journal.ppat.1004058 (PMC4006889; doi:10.1371/journal.ppat.1004058)
Supplement: Text S1 — HCMV constructs utilized and primers used in their generation. A table shows the relevant HCMV viruses as they are referred to in the text, an internal (lab.) BAC reference and any genetic modifications they contain relative to the clinical HCMV Merlin strain. Primer sequences used in their generation are listed, which were used either to target selection cassettes or delete/insert sequences during recombineering reactions or for subsequent sequencing. (DOCX) [file ppat.1004058.s011.docx]

**Text S1**

***HCMV Viruses and Primers Utilzed***

| *Virus Name* | *BAC Ref.* | *Mutations/Deletions* | *GFP/Tags* |
| --- | --- | --- | --- |
| HCMV | pAL 1111 | RL13^-^, UL128^-^ | None |
| ΔUL16 | pAL 1278 | RL13^-^, UL128^-^, ΔUL16, ΔUL18 | UL32-GFP |
| ΔUL142 | pAL 1470 | RL13^-^, UL128^-,^ ΔUL142 | UL32-GFP |
| ΔUS18-22 | pAL 1318 | RL13^-^, UL128^-^, ΔUL16, ΔUL18, ΔUS18-22 | UL32-GFP |
| ΔUS18 | pAL 1654 | RL13^-^, UL128^-^, ΔUS18 | None |
| ΔUS20 | pAL 1595 | RL13^-^, UL128^-^, ΔUS20 | None |
| ΔUS18 & US20 | pAL 1656 | RL13^-^, UL128^-^, ΔUS18, ΔUS20 | None |
| US18-V5 | pAL 1692 | RL13^-^, UL128^-^ | US18-V5 |
| US20-V5 | pAL 1691 | RL13^-^, UL128^-^ | US20-V5 |

***Primer sequences***

*UL32-GFP Primers*

*UL32 Targeting Selection Cassette Forward*

*5’-*CTCCGTCCGTCCTCCTTTCCCGACACGTCACTATCCGATGATTTCATTAAAAAGT

ACGTCTGCGTGTGTGTTTCTTAACCTGTGACGGAAGATCACTTCG-3’

*UL32 Targeting Selection Cassette Reverse*

5’-CATGGGGGGCGCCAAAACGCCGTCGGACGCCGTGCAGAACATCCTCCAAAAGAT

CGAGAAGATTAAGAACACGGAGGAACTGAGGTTCTTATGGCTCTTG-3’

*UL32-GFP Insertion Primer Forward*

5’-ACGCCGTCGGACGCCGTGCAGAACATCCTCCAAAAGATCGAGAAGATTAAGAACA

CGGAGGAAGGATCAGCAGGGTCCGCGATGGTGAGCAAGGGCGAGG-3’

*UL32-GFP Insertion Primer Reverse*

*5’-* TCCGTCCGTCCTCCTTTCCCGACACGTCACTATCCGATGATTTCATTAAAAAGTACG

TCTGCGTGTGTGTTTCTTAATTACTTGTACAGCTCGTCCATGC-3’

*ΔUL16 Primers*

*UL16 Targeting Selection Cassette Forward*

5’-TGCTGACGTAGGTACCGACTGGGGTCAAAAGCCTGGGTACTTATGGGGAGCGCGCAC AAAGGACCGTCAGGCGCCGGCCCTGTGACGGAAGATCACTTCG-3’

*UL16 Targeting Selection Cassette Reverse*

5’-GGAGGGGTGTTTGGCGAGCCCGGATCCGGGCGGTCTCGGATATAGCGAGCC CAATCGGACGTAGAGGCGCTCGATTATTCTGAGGTTCTTATGGCTCTTG-3’

*UL16 Deletion Primer*

5’-AAGCCTGGGTACTTATGGGGAGCGCGCACAAAGGACCGTCAGGCGCCGGCAATAATC GAGCGCCTCTACGTCCGATTGGGCTCGCTATATCCGAGACCGC-3’

*UL16 Sequencing Forward*

5’-GATGTCAAGGAACGCGTGTT-3’

*ΔUL18 Primers*

*UL18 Targeting Selection Cassette Forward*

5’-AAGAAGATAGGAGGGGTCAAAACGCGGACTGAAAGTATATAACGCCGATCATGTTCG AGGAACTGTTAATAAAACGCCCCTGTGACGGAAGATCACTTCG-3’

*UL18 Targeting Selection Cassette Reverse*

5’-CACCGTCCAATTACCGTGTGTAACGCGGCAGGTGTAGTTTTGATTGCAAAAGATGGC TACGTAACATCCCTGATGGAAACTGAGGTTCTTATGGCTCTTG-3’

*UL18 Deletion Primer*

5’-CTGAAAGTATATAACGCCGATCATGTTCGAGGAACTGTTAATAAAACGCCTTTCCATCAG GGATGTTACGTAGCCATCTTTTGCAATCAAAACTACACCT-3’

*UL18 Sequencing Forward*

5’-AGAGGCCCGCCGAGATTC-3’

*ΔUL142 Primers*

*UL142 Targeting Selection Cassette Forward*

5’-ACGATAGGATAATGACCGTTCGCTCCCAACGGATGACACAAAGTATCCGAATAAC

CAACACGCCCATTCAATCCGCATCCTGTGACGGAAGATCACTTCG-3’

*UL142 Targeting Selection Cassette Reverse*

5’-GAGGTGAGAACACGCATAAAATAAAAAAATAAGATGTTAAAAAATGCAGTGTGT

GAAATGTGAATAGTGTGATTAAAATCTGAGGTTCTTATGGCTCTTG-3’

*UL142 Deletion Primer*

5’-AAGTGTGTACTATTTATGGCGGTGTAATTTAGACGGTCTTGCCATCCTGAATTTT

AATCACACTATTCACATTTCACACACTGCATTTTTTAACATCTTA-3’

*UL142 Sequencing Forward*

5’-TTGCATTTGGAGGTGTTGT-3’

*UL142 Sequencing Reverse*

5’-GCGGCATTTTTGGGATTT-3’

*ΔUS18-22 Primers*

*US18-22 Targeting Selection Cassette Forward*

5’-ATGGTGCTGCGCCGAATTGTTAATTAAGGATCCATAACTTCGTATAATG

TATGCTATACGAAGTTATAGCGCTTTTTTACTGAGGTTCTTATGGCTCTTG-3’

*US18-22 Targeting Selection Cassette Reverse*

*5’-* ATGGTGTACAACACGACGCTGACACAGACGCTGTTTTTAGACAACGTTCCACGCTGG TAGATGAGATCCAGGGTCTCGCCTGTGACGGAAGATCACTTCG-3’

*US18-22 Deletion Primer*

5’-TGTAATGCTTTTTACAGGACCGTTCAACAGGTGATACTACCTGCAAGGT

ATAAAAAAGCGCTATAACTTCGTATAGCATACATTATACGAAGTTATGGAT-3’

*US18-22 Sequencing Forward*

*ΔUS18 Primers*

*US18 Targeting Selection Cassette Forward*

5’-GGGAGGTTCATCGTCTGTCTCTAGAGGGAAGGTGGGGAACGTCTAAGCG

AGCGGGAGCGTGTCATCTCCCCCATCTTTCCTGTGACGGAAGATCACTTCG-3’

*US18 Targeting Selection Cassette Reverse*

5’-CGGCCACGTCTGGGTGCAGCAGTACGCCGAGAAACACGGCGGACGCATCG

ACGGCGTGAGTCTCCTCAGCTTGTTGTAACTGAGGTTCTTATGGCTCTTG-3’

*US18 Deletion Primer*

5’-AAGGTGGGGAACGTCTAAGCGAGCGGGAGCGTGTCATCTCCCCCATCTTTG

CTGCCGCTTACGACCGCTGTCGGTCTAAGGTAGGCGTCGATGAAACAGT-3’

*US18 Sequencing Forward*

5’-AGAGTGTAATATAATCACCG-3’

*US18 Sequencing Reverse*

5’-CTCTATGTCGAAAATGTGGC-3’

*US18-V5 Insertion Forward (V5 sequence underlined)*

5’-AGGTGGGGAACGTCTAAGCGAGCGGGAGCGTGTCATCTCCCCCATCTTT

TTACGTAGAATCAAGACCTAGGAGCGGGTTAGGGATTGGCTTACCAGCGCT-3’

*US18-V5 Insertion Reverse (V5 sequence underlined)*

5’-CGAGAAACACGGCGGACGCATCGACGGCGTGAGTCTCCTCAGCTTGTT

GAGCGCTGGTAAGCCAATCCCTAACCCGCTCCTAGGTCTTGATTCTACGTAA-3’

*ΔUS20 Primers*

*US20 Targeting Selection Cassette Forward*

5’-ACGGTCCATTCTAGCGGGACGACATGAAGCATGGCGACAAGCGCGGCTGC

TGTGAAAACGGGCGCGGTTTTATAGGCACCTGTGACGGAAGATCACTTCG-3’

*US20 Targeting Selection Cassette Reverse*

5’-CCGTTGGATTAGTCTTTCGGACGGCGCGCCTTTGGACAACGGGACTTTGAC

AGCCGCCAGTACGACGGGGAAGTCCTAACTGAGGTTCTTATGGCTCTTG-3’

*US20 Deletion Primer*

5’-GCATGGCGACAAGCGCGGCTGCTGTGAAAACGGGCGCGGTTTTATAGGCA

GGTGGCGACGGTCTCGGCACAAAGCCGCTGCGGCGCACCTACCCTTCTCT-3’

*US20 Sequencing Forward*

5’-TAGCTCGGCCACCGGTGGCG-3’

*US20 Sequencing Forward*

5’- TCCGTGCTCTACTTCATGCC-3’

*US20-V5 Insertion Forward (V5 sequence underlined)*

5’-CATGGCGACAAGCGCGGCTGCTGTGAAAACGGGCGCGGTTTTATAGGCA

TTACGTAGAATCAAGACCTAGGAGCGGGTTAGGGATTGGCTTACCAGCGCT-3’

*US20-V5 Insertion Reverse (V5 sequence underlined)*

5’-GCCTTTGGACAACGGGACTTTGACAGCCGCCAGTACGACGGGGAAGTCC

AGCGCTGGTAAGCCAATCCCTAACCCGCTCCTAGGTCTTGATTCTACGTAA-3’
