# Supplementary material for: Two Novel Human Cytomegalovirus NK Cell Evasion Functions Target MICA for Lysosomal Degradation
Source: PLoS Pathog. 2014 May 1;10(5):e1004058. doi: 10.1371/journal.ppat.1004058 (PMC4006889; doi:10.1371/journal.ppat.1004058)
Supplement: Text S2 — Adenovirus constructs utilized and primers used in their generation. A table shows the relevant Adenovirus constructs as they are referred to in the text, an internal (lab.) BAC reference and the gene insert. Primer sequences used in their generation are listed, which were used either to insert the gene sequence during recombineering reactions or for subsequent sequencing. (DOCX) [file ppat.1004058.s012.docx]

***Text S2***

***Adenovirus Constructs and Primers***

| *Virus Name* | *BAC Ref.* | *Gene insert* | *Insert from HCMV strain* | *Tag* |
| --- | --- | --- | --- | --- |
| RAd-CTRL (IE1) | pAL60 | None | N/A- | None |
| RAd-IE1 | pAL31 | IE1 | AD169 | None |
| RAd-CTRL (IE2) | pAL592 | None | N/A | None |
| RAd IE2 | pAL1025 | IE2 | AD169 | None |
| RAd-CTRL | pAL1253 | None | -N/A | (V5) |
| RAd-US18 | mUS18 | US18 | Merlin | V5 |
| RAd-US19 | mUS19 | US19 | Merlin | V5 |
| RAd-US20 | mUS20 | US20 | Merlin | V5 |
| RAd-US21 | mUS21 | US21 | Merlin | V5 |
| RAd-US22 | mUS22 | US22 | Merlin | V5 |

*US18 Forward Primer*

5’-AAGACACCGGGACCGATCCAGCCTGGATCCACAGCGGTCGTAAGCGGCAGCAT-3’

*US18 Reverse Primer*

5’-GAGCGGGTTAGGGATTGGCTTACCAGCGCTCAACAAGCTGAGGAGACTCACGC-3’

*US19 Forward Primer*

5’-AAGACACCGGGACCGATCCAGCCTGGATCCgcgcttgtcgccatgcttca-3’

*US19 Reverse Primer*

5’-GAGCGGGTTAGGGATTGGCTTACCAGCGCTtgggctccacaaccagagcctc-3’

*US20 Forward Primer*

5’-AAGACACCGGGACCGATCCAGCCTGGATCCGAGACCGTCGCCACCATGCAGGCGC-3’

*US20 Reverse Primer*

5’-GAGCGGGTTAGGGATTGGCTTACCAGCGCTGGACTTCCCCGTCGTACTGGCGGCT-3’

*US21 Forward Primer*

5’-AAGACACCGGGACCGATCCAGCCTGGATCCtagacgaatctcggcgataaccg-3’

*US21 Reverse Primer*

5’-GAGCGGGTTAGGGATTGGCTTACCAGCGCTggagacgaactggggcgacg-3’

*US22 Forward Primer*

5’-AAGACACCGGGACCGATCCAGCCTGGATCCagacggctttgccggcatgtc-3’

*US22 Reverse Primer*

5’-GAGCGGGTTAGGGATTGGCTTACCAGCGCTgacccgggtctggtccgtcgt-3’

*2^nd^ Round Amplication Step Common Forward Primer*

5’-AACCGTCAGATCGCCTGGAGACGCCATCCACGCTGTTTTGACCTCCATAGAAGAC

ACCGGGACCGATCCAGCCTGGATCC-3’

2^nd^ Round Amplication Step Common Reverse Primer

5’-GGCGTGACACGTTTATTGAGTAGGATTACAGAGTATAACATAGAGTATAATATAG

AGTATACAATAGTGACGTGGGATCC-3’

*AdZ Sequencing Forward Primer*

5’-AATGTCGTAACAACTCCG-3’

*AdZ Sequencing Reverse Primer*

5’-ACCTGATGGTGATAAGAAG-3’
